# Supplementary material for: Trends and drivers of hypoxic thickness and volume in the northern Gulf of Mexico: 1985–2018
Source: PLoS One. 2024 Dec 5;19(12):e0302759. doi: 10.1371/journal.pone.0302759 (PMC11620451; doi:10.1371/journal.pone.0302759)
Supplement: S1 Text — (DOCX) [file pone.0302759.s001.docx]

**Supporting Information (S1 Text)**

for

**Trends and drivers of hypoxic thickness and volume in the northern Gulf of Mexico:**

**1985-2018**

by

Venkata Rohith Reddy Matli* and Daniel R Obenour

consisting of

14 figures and 10 tables in 30 pages.

*corresponding author, [vmatli@ncsu.edu](mailto:vmatli@ncsu.edu)

# **Summary of monitoring data used in model development.**

Most of the data used in this work were initially collected as a part of study conducted by Matli et al., (2018). Additional data were added to this initial dataset as made available through NCEI (NOAA, 2018; NOAA, 2019; Rabalais, 2019; Rabalais, 2020a; Rabalais, 2020b). Furthermore, data from SEAMAP cruise 1992 were removed due to data integrity issues.

Table 1. Summary of available data

| Source | Number of Observations | Start Date | End Date | Mean Date |
| --- | --- | --- | --- | --- |
| LUMCON | 61 | 7/15/1985 | 7/20/1985 | 7/17/1985 |
| LUMCON | 67 | 7/7/1986 | 7/17/1986 | 7/11/1986 |
| LUMCON | 60 | 7/1/1987 | 7/5/1987 | 7/2/1987 |
| LUMCON | 42 | 8/12/1988 | 8/16/1988 | 8/13/1988 |
| LUMCON | 37 | 8/4/1989 | 8/9/1989 | 8/6/1989 |
| LUMCON | 54 | 7/23/1990 | 7/27/1990 | 7/24/1990 |
| LUMCON | 69 | 7/16/1991 | 7/20/1991 | 7/17/1991 |
| NECOP | 36 | 5/5/1992 | 5/13/1992 | 5/8/1992 |
| NECOP | 65 | 5/14/1992 | 5/20/1992 | 5/17/1992 |
| ~~SEAMAP~~ | ~~43~~ | ~~6/28/1992~~ | ~~7/7/1992~~ | ~~7/3/1992~~ |
| LUMCON | 71 | 7/24/1992 | 7/29/1992 | 7/25/1992 |
| NECOP | 80 | 7/2/1993 | 7/12/1993 | 7/6/1993 |
| LUMCON | 83 | 7/24/1993 | 7/30/1993 | 7/26/1993 |
| SEAMAP | 92 | 7/4/1994 | 7/18/1994 | 7/11/1994 |
| LUMCON | 79 | 7/24/1994 | 7/29/1994 | 7/26/1994 |
| SEAMAP | 85 | 7/6/1995 | 7/18/1995 | 7/13/1995 |
| LUMCON | 75 | 7/21/1995 | 7/26/1995 | 7/23/1995 |
| SEAMAP | 92 | 7/3/1996 | 7/16/1996 | 7/10/1996 |
| LUMCON | 78 | 7/23/1996 | 7/28/1996 | 7/25/1996 |
| SEAMAP | 80 | 6/30/1997 | 7/13/1997 | 7/6/1997 |
| LUMCON | 82 | 7/23/1997 | 7/29/1997 | 7/25/1997 |
| LUMCONT | 9 | 5/12/1998 | 5/12/1998 | 5/12/1998 |
| LDWF | 7 | 5/12/1998 | 5/12/1998 | 5/12/1998 |
| LDWF | 4 | 5/27/1998 | 5/27/1998 | 5/27/1998 |
| LUMCONT | 12 | 6/9/1998 | 6/10/1998 | 6/9/1998 |
| LDWF | 13 | 6/23/1998 | 6/23/1998 | 6/23/1998 |
| SEAMAP | 76 | 7/1/1998 | 7/16/1998 | 7/10/1998 |
| LDWF | 6 | 7/14/1998 | 7/14/1998 | 7/14/1998 |
| LDWF | 12 | 7/21/1998 | 7/21/1998 | 7/21/1998 |
| LUMCON | 84 | 7/16/1998 | 7/26/1998 | 7/22/1998 |
| LDWF | 13 | 8/18/1998 | 8/18/1998 | 8/18/1998 |
| LUMCONT | 10 | 8/11/1998 | 8/19/1998 | 8/18/1998 |
| LDWF | 17 | 8/25/1998 | 8/25/1998 | 8/25/1998 |
| LUMCONT | 9 | 9/22/1998 | 9/22/1998 | 9/22/1998 |
| LDWF | 12 | 9/22/1998 | 9/22/1998 | 9/22/1998 |
| LUMCONT | 9 | 5/4/1999 | 5/4/1999 | 5/4/1999 |
| LDWF | 14 | 5/18/1999 | 5/18/1999 | 5/18/1999 |
| LDWF | 10 | 5/25/1999 | 5/25/1999 | 5/25/1999 |
| LDWF | 18 | 6/3/1999 | 6/3/1999 | 6/3/1999 |
| LUMCONT | 10 | 6/3/1999 | 6/9/1999 | 6/8/1999 |
| LDWF | 10 | 6/17/1999 | 6/30/1999 | 6/19/1999 |
| SEAMAP | 87 | 7/4/1999 | 7/20/1999 | 7/14/1999 |
| LUMCON | 85 | 7/23/1999 | 7/30/1999 | 7/25/1999 |
| LDWF | 14 | 7/29/1999 | 7/29/1999 | 7/29/1999 |
| LDWF | 19 | 8/5/1999 | 8/5/1999 | 8/5/1999 |
| **Source** | **Number of Observations** | **Start Date** | **End Date** | **Mean Date** |
| LUMCONT | 9 | 8/24/1999 | 8/24/1999 | 8/24/1999 |
| LDWF | 13 | 8/31/1999 | 8/31/1999 | 8/31/1999 |
| LDWF | 14 | 9/9/1999 | 9/9/1999 | 9/9/1999 |
| LUMCONT | 10 | 9/7/1999 | 9/14/1999 | 9/13/1999 |
| LUMCONT | 3 | 5/8/2000 | 5/15/2000 | 5/10/2000 |
| LUMCONT | 8 | 5/15/2000 | 5/15/2000 | 5/15/2000 |
| LDWF | 17 | 5/22/2000 | 5/22/2000 | 5/22/2000 |
| LUMCONT | 2 | 6/2/2000 | 6/2/2000 | 6/2/2000 |
| LUMCONT | 9 | 6/14/2000 | 6/14/2000 | 6/14/2000 |
| LDWF | 15 | 6/15/2000 | 6/15/2000 | 6/15/2000 |
| LDWF | 14 | 6/27/2000 | 6/27/2000 | 6/27/2000 |
| LUMCONT | 10 | 7/5/2000 | 7/10/2000 | 7/7/2000 |
| LDWF | 19 | 7/10/2000 | 7/10/2000 | 7/10/2000 |
| SEAMAP | 84 | 7/3/2000 | 7/19/2000 | 7/12/2000 |
| LUMCON | 72 | 7/22/2000 | 7/27/2000 | 7/23/2000 |
| LDWF | 14 | 7/25/2000 | 7/25/2000 | 7/25/2000 |
| LDWF | 17 | 8/9/2000 | 8/9/2000 | 8/9/2000 |
| LUMCONT | 9 | 8/15/2000 | 8/15/2000 | 8/15/2000 |
| LUMCONT | 2 | 8/18/2000 | 8/18/2000 | 8/18/2000 |
| LDWF | 10 | 8/30/2000 | 8/30/2000 | 8/30/2000 |
| LUMCONT | 9 | 9/13/2000 | 9/13/2000 | 9/13/2000 |
| LUMCONT | 16 | 5/7/2001 | 5/8/2001 | 5/7/2001 |
| LDWF | 11 | 5/11/2001 | 5/11/2001 | 5/11/2001 |
| LDWF | 13 | 6/18/2001 | 6/18/2001 | 6/18/2001 |
| LUMCONT | 11 | 6/18/2001 | 6/19/2001 | 6/18/2001 |
| SEAMAP | 66 | 6/13/2001 | 7/25/2001 | 7/10/2001 |
| LDWF | 7 | 7/6/2001 | 7/11/2001 | 7/10/2001 |
| LUMCON | 93 | 7/20/2001 | 7/26/2001 | 7/22/2001 |
| LDWF | 12 | 8/8/2001 | 8/8/2001 | 8/8/2001 |
| LUMCONT | 10 | 8/15/2001 | 8/23/2001 | 8/15/2001 |
| LUMCONT | 2 | 9/6/2001 | 9/6/2001 | 9/6/2001 |
| LUMCONT | 19 | 9/17/2001 | 9/24/2001 | 9/18/2001 |
| LUMCONT | 10 | 5/7/2002 | 5/26/2002 | 5/8/2002 |
| LUMCONT | 19 | 6/8/2002 | 6/12/2002 | 6/11/2002 |
| SEAMAP | 94 | 6/28/2002 | 7/17/2002 | 7/9/2002 |
| LUMCON | 91 | 7/20/2002 | 7/26/2002 | 7/23/2002 |
| LUMCONT | 17 | 8/7/2002 | 8/14/2002 | 8/13/2002 |
| LUMCONT | 15 | 8/26/2002 | 8/29/2002 | 8/27/2002 |
| LUMCONT | 9 | 9/20/2002 | 9/20/2002 | 9/20/2002 |
| LUMCONT | 17 | 5/13/2003 | 5/14/2003 | 5/13/2003 |
| LUMCONT | 3 | 6/4/2003 | 6/9/2003 | 6/5/2003 |
| EPA | 41 | 6/12/2003 | 6/19/2003 | 6/15/2003 |
| LUMCONT | 10 | 6/16/2003 | 6/16/2003 | 6/16/2003 |
| SEAMAP | 53 | 7/3/2003 | 7/28/2003 | 7/14/2003 |
| LUMCON | 92 | 7/23/2003 | 7/28/2003 | 7/25/2003 |
| UMCES | 19 | 7/30/2003 | 8/4/2003 | 8/2/2003 |
| LUMCONT | 8 | 8/14/2003 | 8/15/2003 | 8/14/2003 |
| LUMCONT | 10 | 8/22/2003 | 8/22/2003 | 8/22/2003 |
| LUMCONT | 9 | 9/9/2003 | 9/9/2003 | 9/9/2003 |
| TAMU | 37 | 9/13/2003 | 9/16/2003 | 9/14/2003 |
| LUMCONT | 2 | 5/5/2004 | 5/5/2004 | 5/5/2004 |
| LUMCONT | 9 | 5/13/2004 | 5/13/2004 | 5/13/2004 |
| LUMCONT | 2 | 6/6/2004 | 6/6/2004 | 6/6/2004 |
| LUMCONT | 16 | 6/15/2004 | 6/16/2004 | 6/15/2004 |
| TAMU | 60 | 6/26/2004 | 7/1/2004 | 6/28/2004 |
| **Source** | **Number of Observations** | **Start Date** | **End Date** | **Mean Date** |
| SEAMAP | 96 | 6/28/2004 | 7/14/2004 | 7/8/2004 |
| LUMCON | 84 | 7/21/2004 | 7/26/2004 | 7/23/2004 |
| UMCES | 15 | 7/28/2004 | 8/1/2004 | 7/30/2004 |
| LUMCONT | 9 | 8/19/2004 | 8/19/2004 | 8/19/2004 |
| TAMU | 64 | 8/20/2004 | 8/26/2004 | 8/23/2004 |
| LUMCONT | 18 | 8/30/2004 | 9/9/2004 | 9/8/2004 |
| LUMCONT | 9 | 5/11/2005 | 5/11/2005 | 5/11/2005 |
| TAMU | 102 | 5/20/2005 | 5/26/2005 | 5/23/2005 |
| LUMCONT | 16 | 6/2/2005 | 6/3/2005 | 6/2/2005 |
| TAMU | 76 | 7/8/2005 | 7/14/2005 | 7/11/2005 |
| SEAMAP | 41 | 7/4/2005 | 7/16/2005 | 7/12/2005 |
| LUMCON | 81 | 7/25/2005 | 7/30/2005 | 7/26/2005 |
| SEAMAP | 34 | 7/28/2005 | 7/31/2005 | 7/29/2005 |
| LUMCONT | 16 | 8/16/2005 | 8/18/2005 | 8/16/2005 |
| TAMU | 126 | 8/18/2005 | 8/24/2005 | 8/21/2005 |
| LUMCONT | 9 | 9/7/2005 | 9/7/2005 | 9/7/2005 |
| LUMCONT | 41 | 9/28/2005 | 9/30/2005 | 9/28/2005 |
| LUMCONT | 9 | 5/26/2006 | 5/27/2006 | 5/26/2006 |
| LUMCONT | 4 | 6/6/2006 | 6/6/2006 | 6/6/2006 |
| EPA | 121 | 6/6/2006 | 6/18/2006 | 6/11/2006 |
| LUMCONT | 17 | 6/26/2006 | 6/27/2006 | 6/26/2006 |
| SEAMAP | 94 | 6/29/2006 | 7/16/2006 | 7/10/2006 |
| LUMCON | 87 | 7/21/2006 | 7/27/2006 | 7/24/2006 |
| UMCES | 40 | 8/4/2006 | 8/13/2006 | 8/9/2006 |
| LUMCONT | 8 | 9/11/2006 | 9/11/2006 | 9/11/2006 |
| EPA | 127 | 9/6/2006 | 9/18/2006 | 9/11/2006 |
| EPA | 83 | 5/1/2007 | 5/7/2007 | 5/4/2007 |
| LUMCONT | 16 | 5/12/2007 | 5/13/2007 | 5/12/2007 |
| LUMCONT | 9 | 6/6/2007 | 6/6/2007 | 6/6/2007 |
| TAMU | 63 | 7/17/2007 | 7/20/2007 | 7/18/2007 |
| SEAMAP | 66 | 6/25/2007 | 8/3/2007 | 7/22/2007 |
| LUMCON | 94 | 7/22/2007 | 7/28/2007 | 7/24/2007 |
| UMCES | 60 | 7/30/2007 | 8/7/2007 | 8/3/2007 |
| LUMCONT | 9 | 8/15/2007 | 8/15/2007 | 8/15/2007 |
| EPA | 130 | 8/19/2007 | 8/31/2007 | 8/23/2007 |
| TAMU | 74 | 9/6/2007 | 9/10/2007 | 9/7/2007 |
| LUMCONT | 16 | 9/11/2007 | 9/12/2007 | 9/11/2007 |
| LUMCONT | 9 | 5/12/2008 | 5/13/2008 | 5/12/2008 |
| LUMCONT | 16 | 6/11/2008 | 6/13/2008 | 6/11/2008 |
| SEAMAP | 102 | 6/29/2008 | 7/16/2008 | 7/9/2008 |
| TAMU | 72 | 7/17/2008 | 7/20/2008 | 7/18/2008 |
| LUMCON | 89 | 7/21/2008 | 7/28/2008 | 7/24/2008 |
| UMCES | 73 | 8/1/2008 | 8/12/2008 | 8/7/2008 |
| LUMCONT | 4 | 8/15/2008 | 8/15/2008 | 8/15/2008 |
| LUMCONT | 9 | 5/26/2009 | 5/29/2009 | 5/26/2009 |
| LUMCONT | 8 | 6/12/2009 | 6/12/2009 | 6/12/2009 |
| SEAMAP | 167 | 6/23/2009 | 7/15/2009 | 7/5/2009 |
| LUMCON | 93 | 7/18/2009 | 7/23/2009 | 7/20/2009 |
| TAMU | 29 | 7/28/2009 | 7/31/2009 | 7/29/2009 |
| LUMCONT | 16 | 8/10/2009 | 8/11/2009 | 8/10/2009 |
| LUMCONT | 2 | 8/24/2009 | 8/25/2009 | 8/24/2009 |
| LUMCONT | 7 | 9/21/2009 | 9/21/2009 | 9/21/2009 |
| SEAMAP | 77 | 7/13/2010 | 8/2/2010 | 7/24/2010 |
| LUMCON | 90 | 7/25/2010 | 7/31/2010 | 7/28/2010 |
| UMCES | 30 | 9/2/2010 | 9/7/2010 | 9/4/2010 |
| **Source** | **Number of Observations** | **Start Date** | **End Date** | **Mean Date** |
| LUMCONT | 15 | 5/16/2011 | 5/30/2011 | 5/23/2011 |
| LUMCONT | 15 | 6/17/2011 | 6/18/2011 | 6/17/2011 |
| TAMU | 48 | 6/24/2011 | 6/28/2011 | 6/26/2011 |
| SEAMAP | 95 | 7/2/2011 | 7/16/2011 | 7/10/2011 |
| LUMCON | 89 | 7/24/2011 | 7/30/2011 | 7/26/2011 |
| LUMCONT | 7 | 8/22/2011 | 8/23/2011 | 8/22/2011 |
| LUMCONT | 15 | 5/1/2012 | 5/2/2012 | 5/1/2012 |
| TAMU | 46 | 6/11/2012 | 6/15/2012 | 6/13/2012 |
| LUMCONT | 15 | 6/16/2012 | 6/17/2012 | 6/16/2012 |
| SEAMAP | 76 | 6/16/2012 | 6/29/2012 | 6/23/2012 |
| LUMCON | 66 | 7/22/2012 | 7/27/2012 | 7/24/2012 |
| TAMU | 49 | 8/16/2012 | 8/20/2012 | 8/17/2012 |
| LUMCONT | 10 | 8/16/2012 | 8/22/2012 | 8/21/2012 |
| SEAMAP | 66 | 6/16/2013 | 6/30/2013 | 6/22/2013 |
| LUMCON | 99 | 7/21/2013 | 7/28/2013 | 7/24/2013 |
| LUMCONT | 1 | 5/26/2014 | 5/26/2014 | 5/26/2014 |
| SEAMAP | 119 | 6/17/2014 | 7/5/2014 | 6/27/2014 |
| LUMCON | 85 | 7/27/2014 | 8/2/2014 | 7/30/2014 |
| SEAMAP | 94 | 6/17/2015 | 7/5/2015 | 6/27/2015 |
| SEAMAP | 138 | 6/16/2016 | 7/3/2016 | 6/25/2016 |
| SEAMAP | 70 | 6/16/2017 | 7/01/2017 | 6/25/2017 |
| LUMCON | 90 | 7/23/2017 | 7/29/2017 | 7/26/2017 |
| SEAMAP | 79 | 6/20/2018 | 7/25/2018 | 7/05/2018 |
| LUMCON | 79 | 7/23/2018 | 7/27/2018 | 7/25/2018 |
| SEAMAP | 78 | 6/14/2019 | 7/03/2019 | 6/23/2019 |

References

1. Matli, V. R. R., Fang, S., Guinness, J., Rabalais, N. N., Craig, J. K., & Obenour, D. R. (2018). Space-time geostatistical assessment of hypoxia in the northern Gulf of Mexico. *Environmental Science & Technology*, *52*(21), 12484-12493.
2. NOAA National Marine Fisheries Service; NOAA National Centers for Environmental Information (2019). Water temperature, salinity, dissolved oxygen, and other measurements from CTD taken from NOAA Ship Oregon II in the Gulf of Mexico from 2019-06-09 to 2019-07-18 as part of the Southeast Area Monitoring and Assessment Program (SEAMAP) (NCEI Accession 0193188). NOAA National Centers for Environmental Information. Dataset. https://www.ncei.noaa.gov/archive/accession/0193188. Accessed [07/06/2020].
3. NOAA, National Marine Fisheries Service; US DOC, NOAA, NESDIS, National Centers for Environmental Information (2018). Water temperature, salinity, dissolved oxygen, and other measurements from CTD taken from NOAA Ship Oregon II in the Gulf of Mexico from 2018-06-10 to 2018-07-19 as part of the Southeast Area Monitoring and Assessment Program (SEAMAP) (NCEI Accession 0174810). NOAA National Centers for Environmental Information. Dataset. https://www.ncei.noaa.gov/archive/accession/0174810. Accessed [07/06/2020].
4. Rabalais, Nancy (2019). Water temperature, salinity, and other physical, chemical, and biological parameters taken by CTD and multi parameter water quality sonde on board of research vessel Pelican on the Texas-Louisiana continental shelf, Gulf of Mexico from 2015-06-23 to 2015-12-09 (NCEI Accession 0205844). NOAA National Centers for Environmental Information. Dataset. https://doi.org/10.25921/rgcz-5338. Accessed [07/06/2020].
5. Rabalais, Nancy (2020a). Physical (hydrography), chemical (CTD), and biological (water quality) processes of the Texas-Louisiana continental shelf, 2017 (NCEI Accession 0208325). [indicate subset used]. NOAA National Centers for Environmental Information. Dataset. https://accession.nodc.noaa.gov/0208325. Accessed [07/06/2020]
6. Rabalais, Nancy (2020b). Physical (hydrography), chemical (CTD), and biological (water quality) processes of the Texas-Louisiana continental shelf, 2018 (NCEI Accession 0219157). [indicate subset used]. NOAA National Centers for Environmental Information. Dataset. https://www.ncei.noaa.gov/archive/accession/0219157. Accessed [07/06/2020].

# **Histograms of observed hypoxic thickness and fraction**


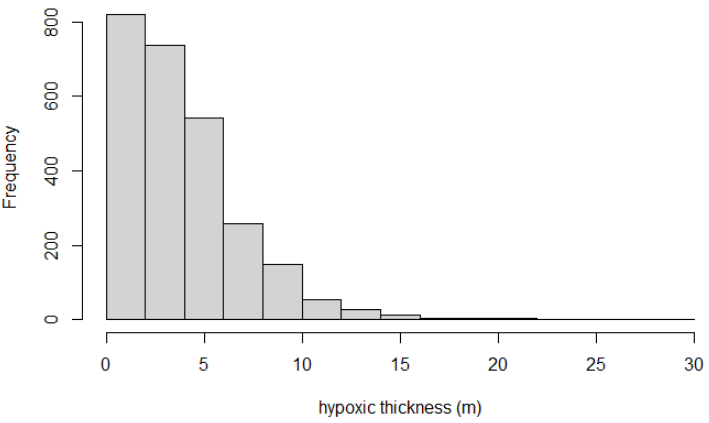

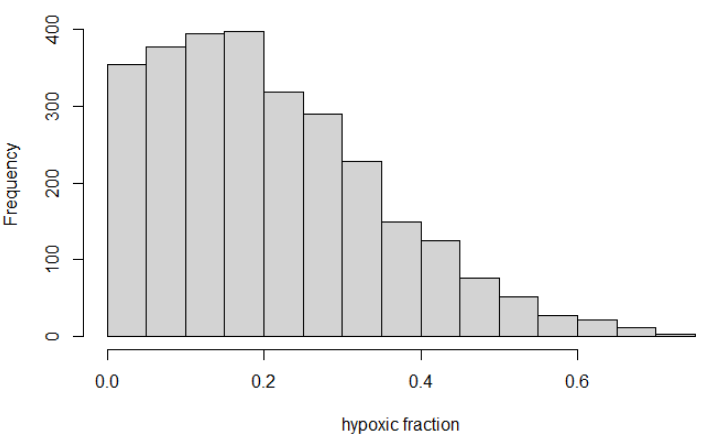

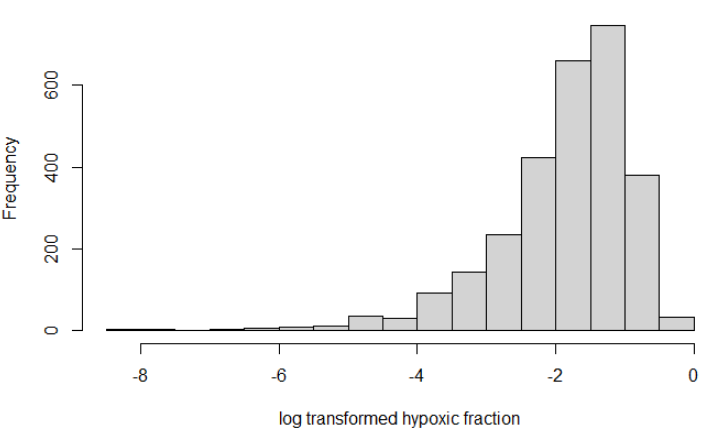

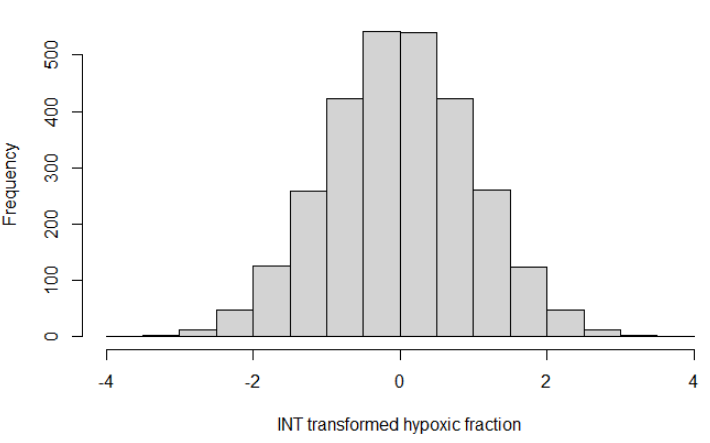


Figure 1. Histograms of hypoxic thickness (top left), hypoxic fraction (top right), log-transformed hypoxic fraction (bottom left), and INT transformed hypoxic fraction (bottom right)

# **Instrument bias adjustment**

Our monitoring data come from various instruments and organizations. While a majority of cruises use rosette-mounted DO samplers, there are a significant number of samples collected using handheld instruments. The difference between these methods is the depth to which the DO measurements are typically collected. Handheld samplers are versatile and capable of collecting samples at the sea floor. Rosette-mounted samplers, however, are on large frames with other sensors. To avoid damage, rosette-mounted samplers do not sample all the way to the sea floor.

To address the bias associated with Rosette sampling, a correction factor was determined from sampling events that used both rosette and handheld sensors in parallel. Observations sampled using both instruments were first divided into two categories based on the difference in BWDO collected using both the sensors. The first category comprises of observations with the difference in BWDO values between both the instruments less than 2 times the standard deviation of uncorrelated stochasticity (square root of nugget of the geostatistical model). The second group comprise of all the remaining observations of BWDO (where the difference is greater than 2 times the square root of nugget). These groups (1 and 2) constitute 88.9% and 11.1% of the observed data, respectively.

Groups one and two are modelled using equations (1) and (2) respectively. A more detailed procedure of determining these equations is described in Obenour et al., (2013). Samples from rosette-only sensor are corrected using Eq. (1) at 88.9% and Eq. (2) at 11.1% probability (corresponding to the probabilities in the observed data, noted above). All rosette-only measures of HF are corrected using Eq. (3). These correction factors are derived from Obenour et al., 2013.

BWDO_Adj_ = 0.973 × BWDO_Rosette_ + 𝜖 …………………..…….Eq (1)

BWDO_Adj_ = U(0,[BWDO_Rosette_ – 2($\sigma_{\varepsilon}$)]) ………....……...Eq (2)

HT_Adj_ = 0.82 + HT_Rosette_ + 𝜖, where 𝜖 ~ N(0,0.36) ………..……..Eq (3)

The preceding equations are based on the rosette-mounted sampler reaching about 1 meter from the sea floor. However, there was evidence that a small portion of sampling events missed the sea floor by a larger margin. This can potentially result of a faulty depth sensor or operator error. Cruises were identified to be faulty if they meet all of the following three criteria: (a) shelfwide cruise with more than 10 samples; (b) more than 10% of the events are under-sampled; (c) the mean difference between maximum instrument depth and ocean depth is greater than 3 meters across all samples. Ocean depth corresponding to samples is determined by overlaying sample locations with bathymetry raster from digital elevation models generated using coastal relief model (National Geophysical Data Center, 2001). Sampling events are considered to be under-sampled if the difference between ocean depth and instrument depth is greater than or equal to 5 meters in shallow regions (ocean depth<=15 meters), or if the instrument did not reach to the bottom fifth of the water column in deep regions (ocean depth>15 meters). There were four cruises that required this additional bias correction (2 NECOP cruises in May 1992, 1 NECOP cruise in July 1993, and 1 SEAMAP cruise in July 1994), in addition to half of the observations from the LUMCON cruise in 1991 (Obenour et al., 2013). For these cruises, BWDO and HT (BWDO_ErCr_, HT_ErCr_) were corrected using Eq. (4), (5), and (6). Additional details on the development of these equations are provided in Obenour et al., (2013).

BWDO_CruAdj_ = BWDO_ErCr_ × 0.967 – 0.163 + 𝜖 ………...……Eq (4)

BWDO_CruAdj_ = U(0,[ BWDO_ErCr_ – 2($\sigma_{\varepsilon})$]) ………...……Eq (5)

HT_CruAdj_ = HT_ErCr_ + 2.3492 + 𝜖, where 𝜖 ~ N(0,0.39) ……….….Eq (6)

For BWDO observations that need this additional adjustment, Eq. (4) was applied at a 67.5% probability and Eq. (5) was applied at a 32.5% probability (consistent with observed data).

These correction factors were applied to observations when performing conditional realizations. Adjusted HT values were converted to the transformed scale using the equations shown in Fig. 1 of the manuscript.

Reference

1. National Geophysical Data Center, 2001. U.S. Coastal Relief Model - Central Gulf of Mexico. National Geophysical Data Center, NOAA. doi:10.7289/V54Q7RW0 [access date: 07/06/2019].

# **Additional parameters and trends of BWDO and HF_t_ models.**

Table 2. Annual intercept values for BWDO and HF_t_ models

| \| Year \| BWDO \| \| \| --- \| --- \| --- \| \| *β* \| *σ_β_* \| \| 1985 \| 5.04 \| 0.58 \| \| 1986 \| 5.14 \| 0.64 \| \| 1987 \| 5.55 \| 0.67 \| \| 1988 \| 7.87 \| 0.75 \| \| 1989 \| 5.87 \| 0.82 \| \| 1990 \| 5.48 \| 0.64 \| \| 1991 \| 5.32 \| 0.65 \| \| 1992 \| 5.66 \| 0.35 \| \| 1993 \| 4.76 \| 0.38 \| \| 1994 \| 5.62 \| 0.42 \| \| 1995 \| 4.78 \| 0.50 \| \| 1996 \| 4.81 \| 0.48 \| \| 1997 \| 4.35 \| 0.47 \| \| 1998 \| 5.37 \| 0.38 \| \| 1999 \| 5.13 \| 0.38 \| \| 2000 \| 6.27 \| 0.38 \| \| 2001 \| 5.77 \| 0.37 \| \| 2002 \| 5.57 \| 0.37 \| \| 2003 \| 5.67 \| 0.34 \| \| 2004 \| 5.15 \| 0.35 \| \| 2005 \| 5.20 \| 0.34 \| \| 2006 \| 5.63 \| 0.32 \| \| 2007 \| 5.31 \| 0.33 \| \| 2008 \| 4.64 \| 0.39 \| \| 2009 \| 5.71 \| 0.38 \| \| 2010 \| 5.21 \| 0.41 \| \| 2011 \| 4.87 \| 0.37 \| \| 2012 \| 5.52 \| 0.35 \| \| 2013 \| 5.38 \| 0.43 \| \| 2014 \| 5.50 \| 0.41 \| \| 2015 \| 5.63 \| 0.41 \| \| 2016 \| 4.58 \| 0.49 \| \| 2017 \| 4.89 \| 0.43 \| \| 2018 \| 5.79 \| 0.44 \| \| 2019 \| 4.89 \| 0.51 \| | \| Year \| HF_t_ \| \| \| --- \| --- \| --- \| \| *β* \| *σ_β_* \| \| 1985 \| 0.33 \| 0.28 \| \| 1986 \| 0.18 \| 0.26 \| \| 1987 \| -0.12 \| 0.28 \| \| 1988 \| -0.46 \| 0.85 \| \| 1989 \| -0.39 \| 0.34 \| \| 1990 \| -0.10 \| 0.26 \| \| 1991 \| -0.04 \| 0.24 \| \| 1992 \| 0.03 \| 0.19 \| \| 1993 \| 0.30 \| 0.17 \| \| 1994 \| 0.27 \| 0.18 \| \| 1995 \| 0.44 \| 0.19 \| \| 1996 \| 0.67 \| 0.18 \| \| 1997 \| 0.44 \| 0.18 \| \| 1998 \| 0.18 \| 0.18 \| \| 1999 \| 0.60 \| 0.16 \| \| 2000 \| 0.62 \| 0.23 \| \| 2001 \| 0.44 \| 0.17 \| \| 2002 \| 0.18 \| 0.16 \| \| 2003 \| 0.11 \| 0.17 \| \| 2004 \| 0.46 \| 0.14 \| \| 2005 \| 0.36 \| 0.15 \| \| 2006 \| 0.27 \| 0.15 \| \| 2007 \| 0.42 \| 0.14 \| \| 2008 \| 0.35 \| 0.15 \| \| 2009 \| 0.58 \| 0.18 \| \| 2010 \| 0.10 \| 0.19 \| \| 2011 \| 0.43 \| 0.15 \| \| 2012 \| 0.45 \| 0.17 \| \| 2013 \| 0.62 \| 0.21 \| \| 2014 \| 0.25 \| 0.19 \| \| 2015 \| 0.67 \| 0.20 \| \| 2016 \| 1.05 \| 0.24 \| \| 2017 \| 0.63 \| 0.20 \| \| 2018 \| 0.64 \| 0.24 \| \| 2019 \| 0.78 \| 0.30 \| |
| --- | --- | --- | --- | --- | --- | --- | --- | --- | --- | --- | --- | --- | --- | --- | --- | --- | --- | --- | --- | --- | --- | --- | --- | --- | --- | --- | --- | --- | --- | --- | --- | --- | --- | --- | --- | --- | --- | --- | --- | --- | --- | --- | --- | --- | --- | --- | --- | --- | --- | --- | --- | --- | --- | --- | --- | --- | --- | --- | --- | --- | --- | --- | --- | --- | --- | --- | --- | --- | --- | --- | --- | --- | --- | --- | --- | --- | --- | --- | --- | --- | --- | --- | --- | --- | --- | --- | --- | --- | --- | --- | --- | --- | --- | --- | --- | --- | --- | --- | --- | --- | --- | --- | --- | --- | --- | --- | --- | --- | --- | --- | --- | --- | --- | --- | --- | --- | --- | --- | --- | --- | --- | --- | --- | --- | --- | --- | --- | --- | --- | --- | --- | --- | --- | --- | --- | --- | --- | --- | --- | --- | --- | --- | --- | --- | --- | --- | --- | --- | --- | --- | --- | --- | --- | --- | --- | --- | --- | --- | --- | --- | --- | --- | --- | --- | --- | --- | --- | --- | --- | --- | --- | --- | --- | --- | --- | --- | --- | --- | --- | --- | --- | --- | --- | --- | --- | --- | --- | --- | --- | --- | --- | --- | --- | --- | --- | --- | --- | --- | --- | --- | --- | --- | --- | --- | --- | --- | --- | --- | --- | --- | --- | --- | --- | --- | --- | --- | --- | --- | --- | --- | --- |

*Note*: The coefficients associated with HF_t_ are unitless because fraction is a unitless value. The coefficients associated with BWDO model are (mg/L)


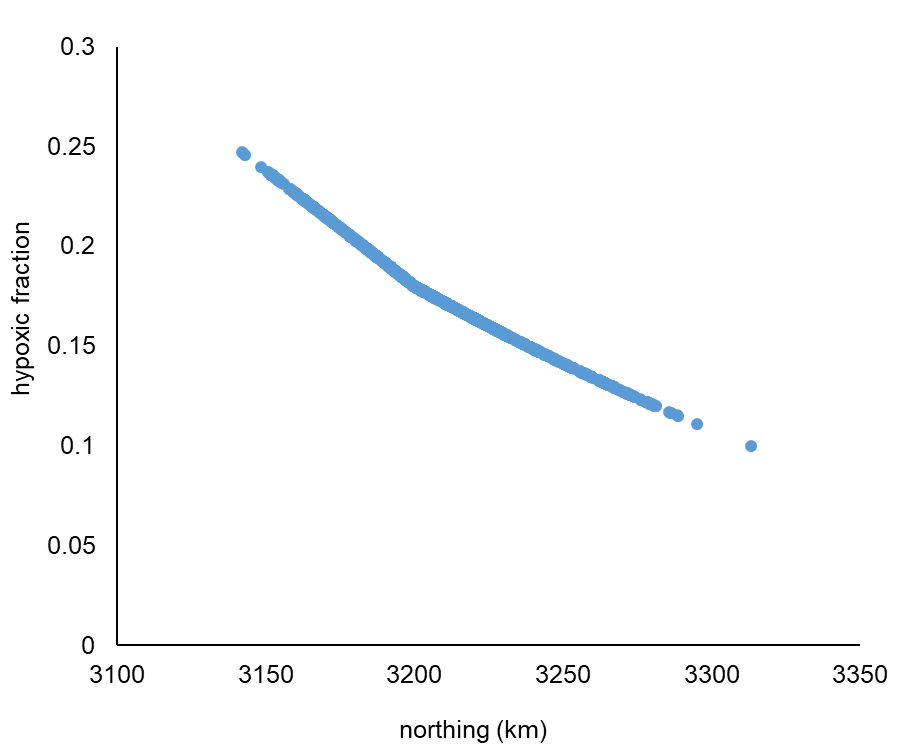

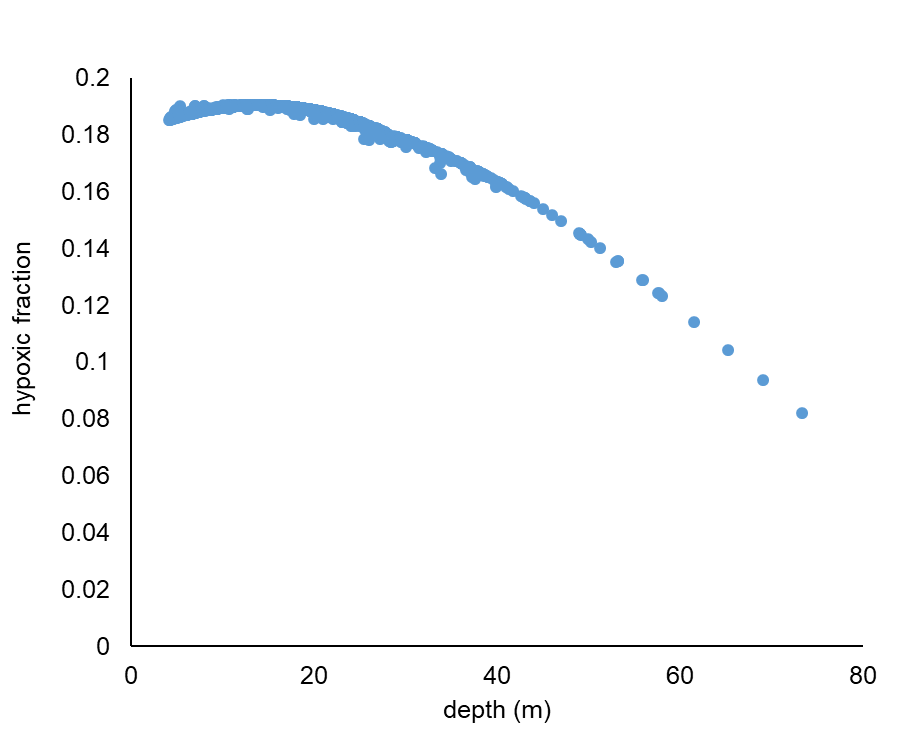

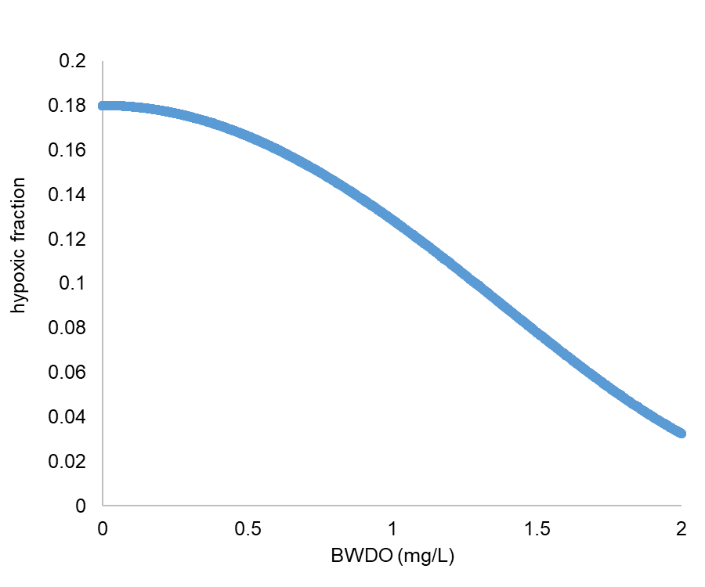


Figure 2. Effects of BIC-selected trend variables on HF (blue dots correspond to observed data).

Table 3. Geostatistical covariance function parameters $\sigma_{\varepsilon}^{2}$(nugget), $\sigma_{\eta}^{2}$ (partial sill), *α* (spatial anisotropy ratio), *a* (spatial range parameter), *b* (temporal range parameter) for HF_t_ and BWDO models.

| Variable | $\boldsymbol{\sigma}_{\boldsymbol{\varepsilon}}^{\boldsymbol{2}}$ | $\boldsymbol{\sigma}_{\boldsymbol{\eta}}^{\boldsymbol{2}}$ | *α* | a (km) | *b* (days) |
| --- | --- | --- | --- | --- | --- |
| BWDO (mg/L) | 0.47 | 2.58 | 2.26 | 64 | 9 |
| HF_t_ (unitless) | 0.20 | 0.52 | 1.62 | 23 | 4 |

*Note*: Units of partial sill and nugget are the square of the units of variable being considered. Spatial and temporal ranges are approximately three times the associated range parameters.

# **Trend maps of hypoxic fraction and thickness**


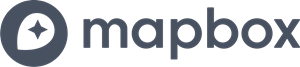

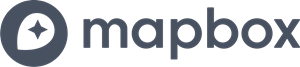

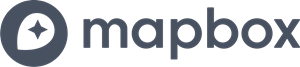

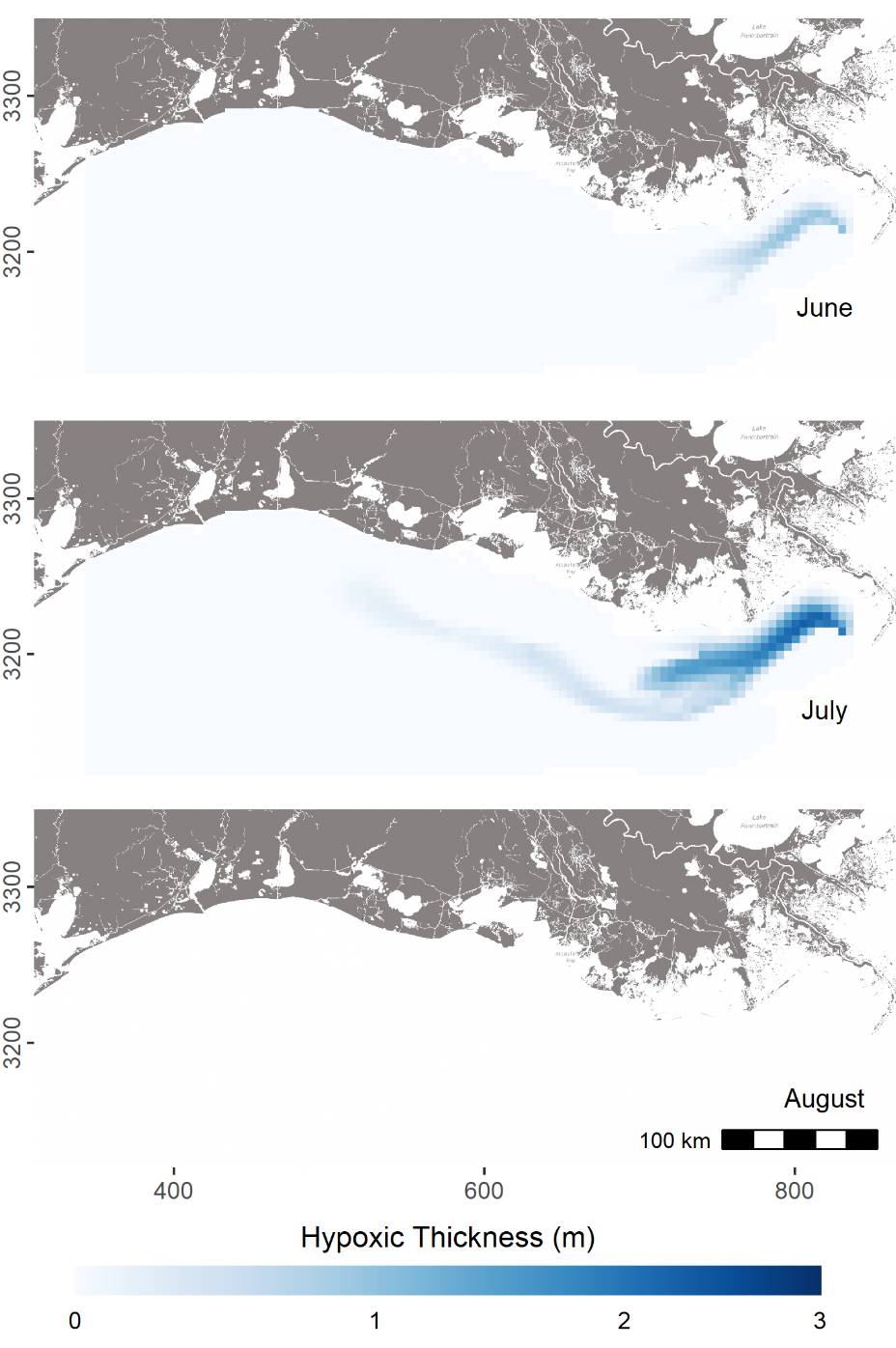

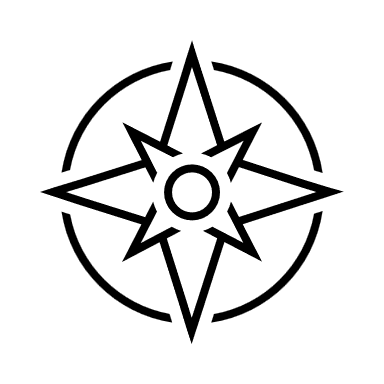

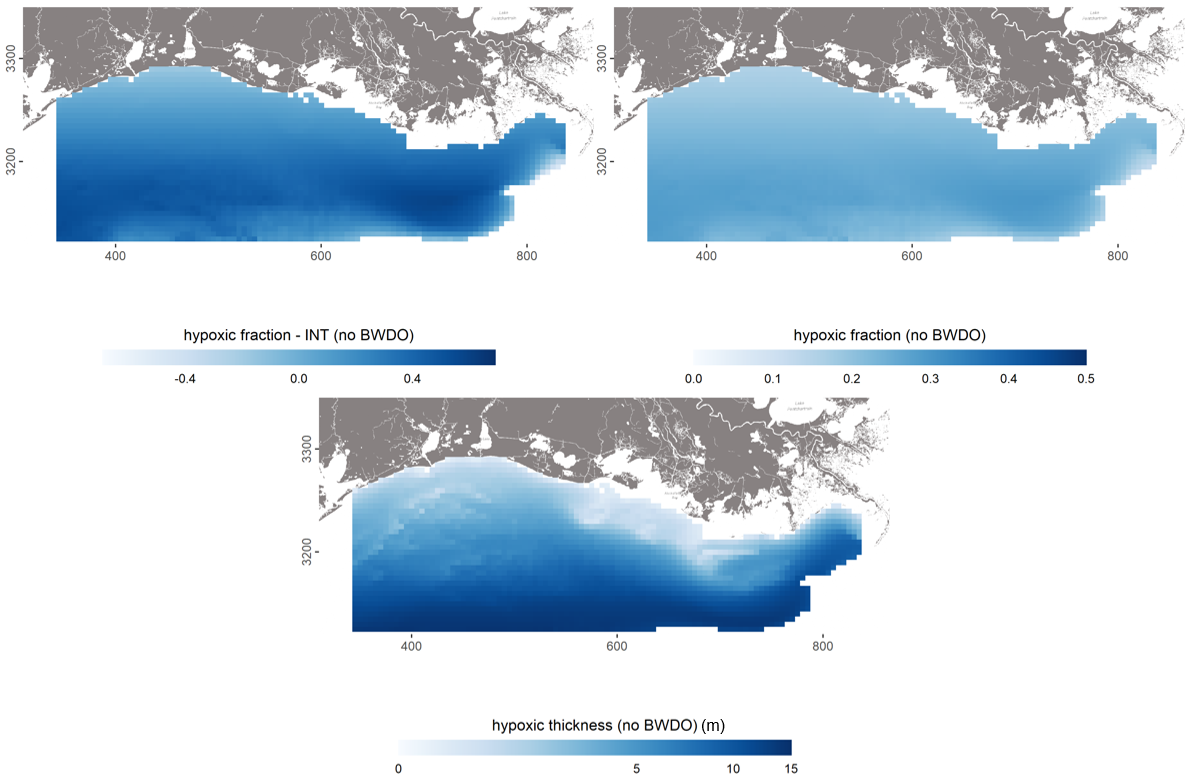

Figure 3. Mapped effects of trends with northing and depth on INT transformed HF (top left), HF (top right) and HT (bottom) in an average year. BWDO is assumed constant across the shelf at zero mg/L.

© Mapbox © OpenStreetMap

© Mapbox © OpenStreetMap

© Mapbox © OpenStreetMap


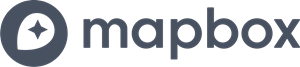

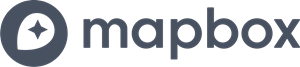

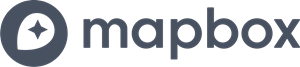

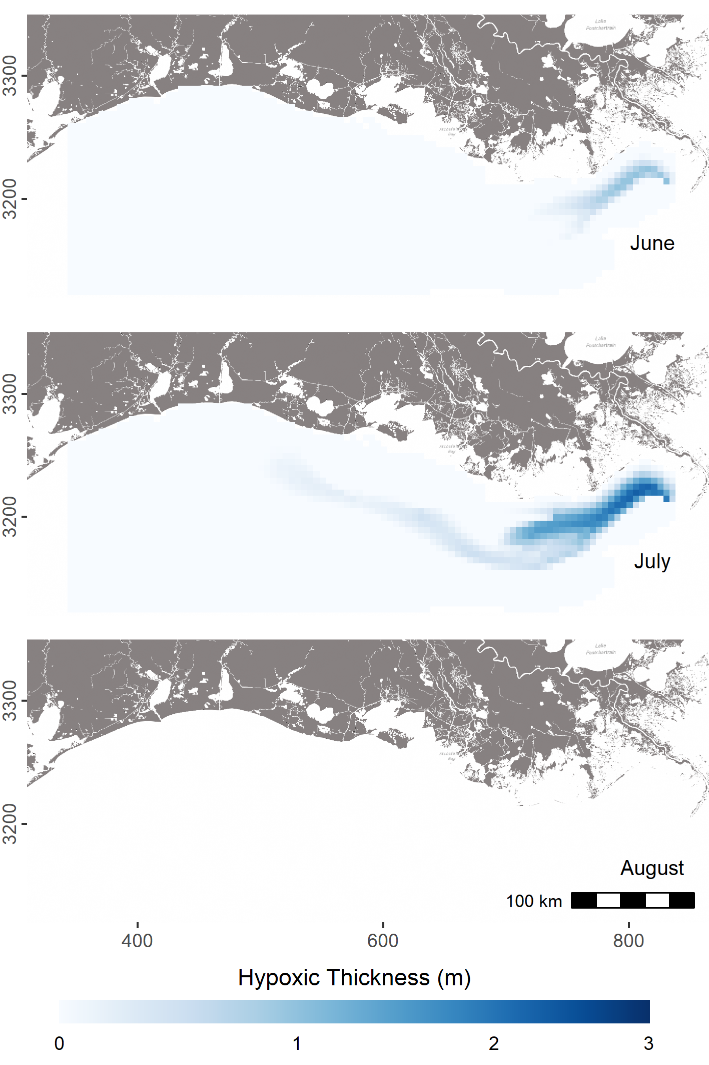

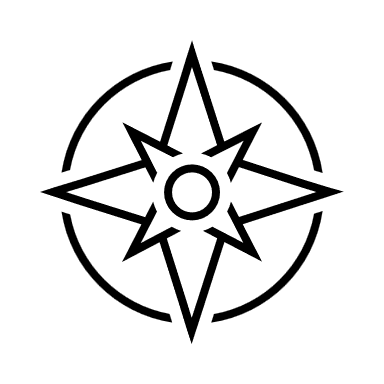

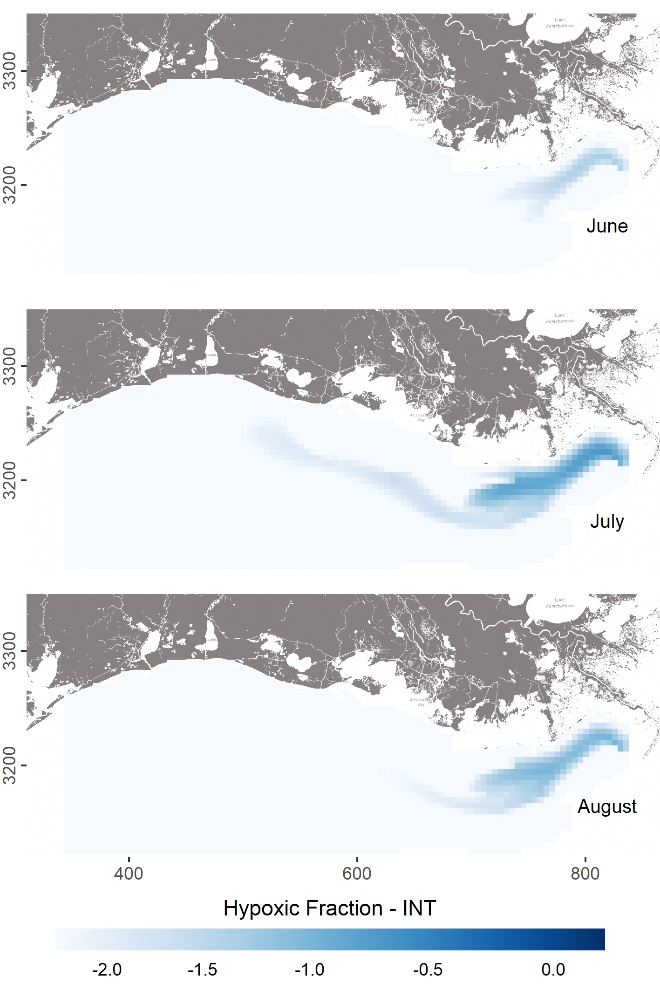

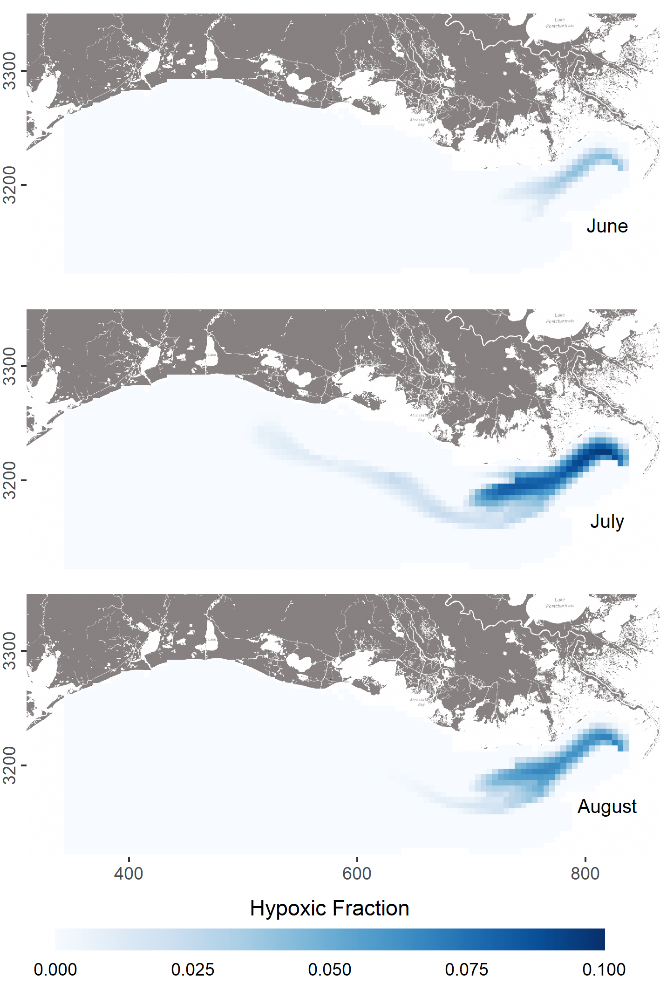

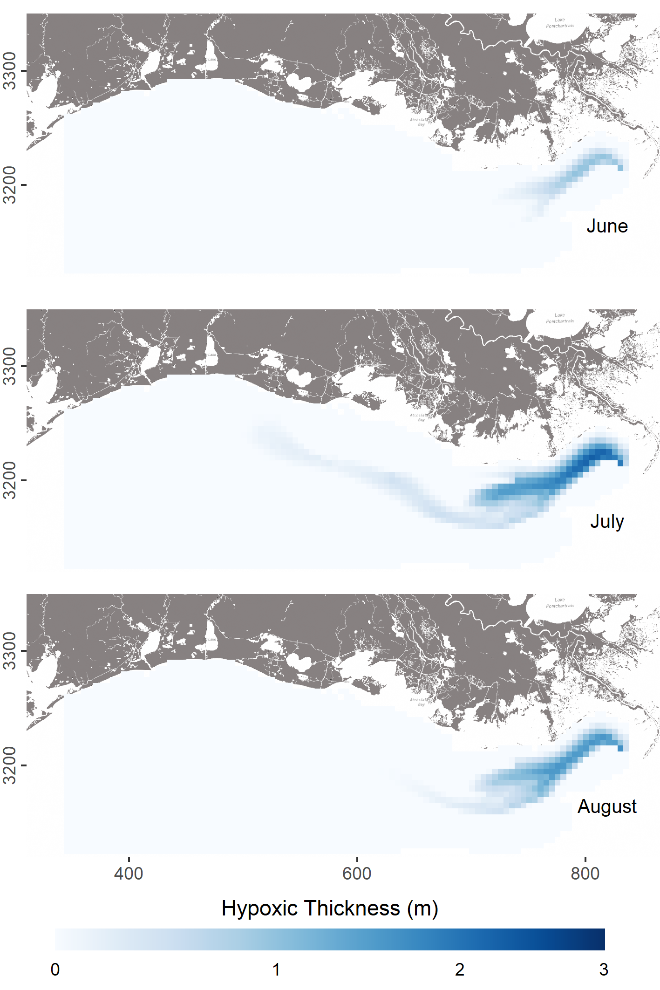


© Mapbox © OpenStreetMap

© Mapbox © OpenStreetMap

© Mapbox © OpenStreetMap

Figure 4. Mapped effects of trends with northing, depth, and BWDO on INT transformed HF (left), HF (center), and HT (right) in an average year in months of June, July, and August

# **Daily estimates of hypoxia from geostatistical model**


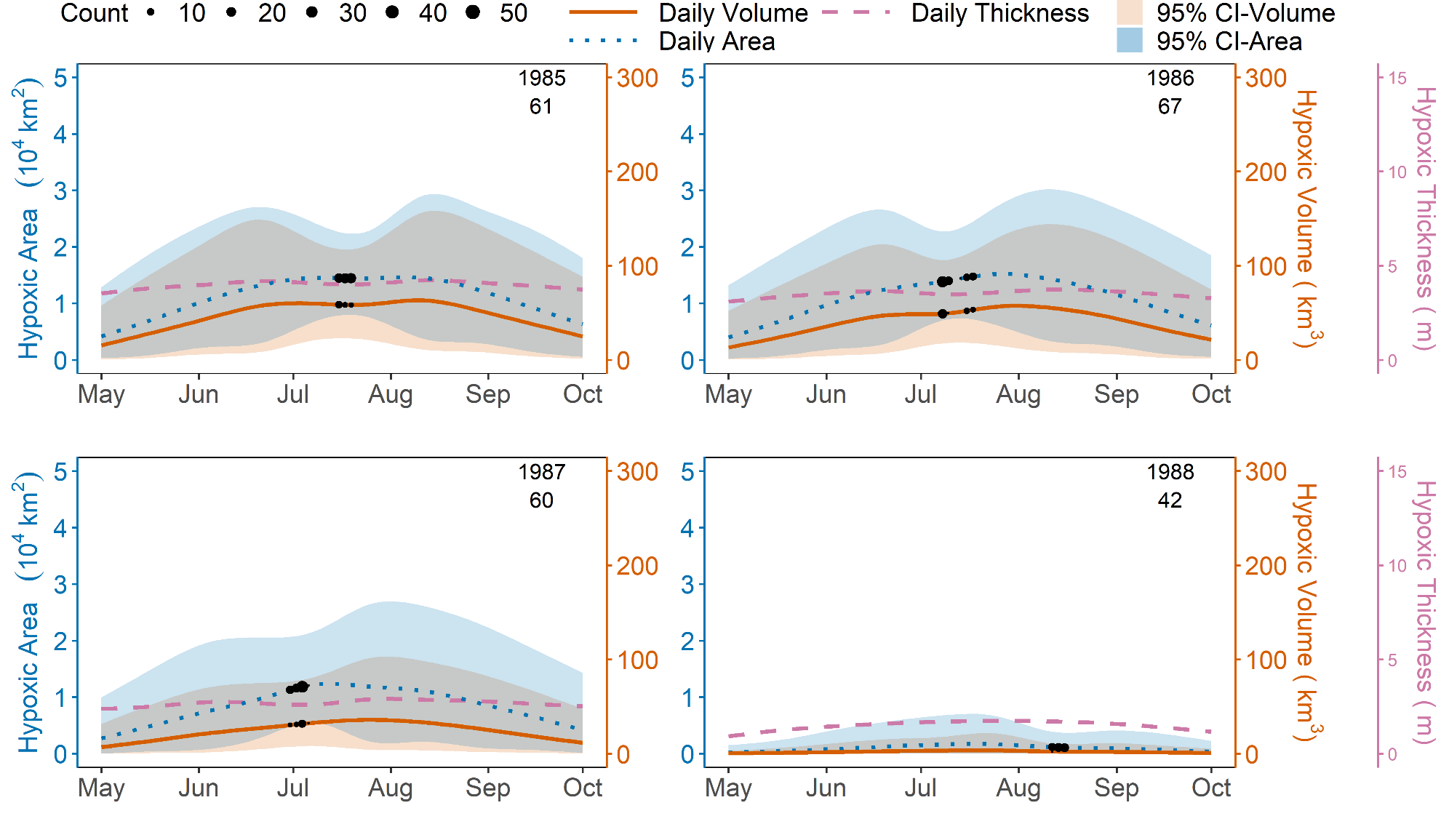


Figure 5. Summer-wide daily estimates of area, volume, and thickness with the 95% CI of area, and volume for 1985-1988


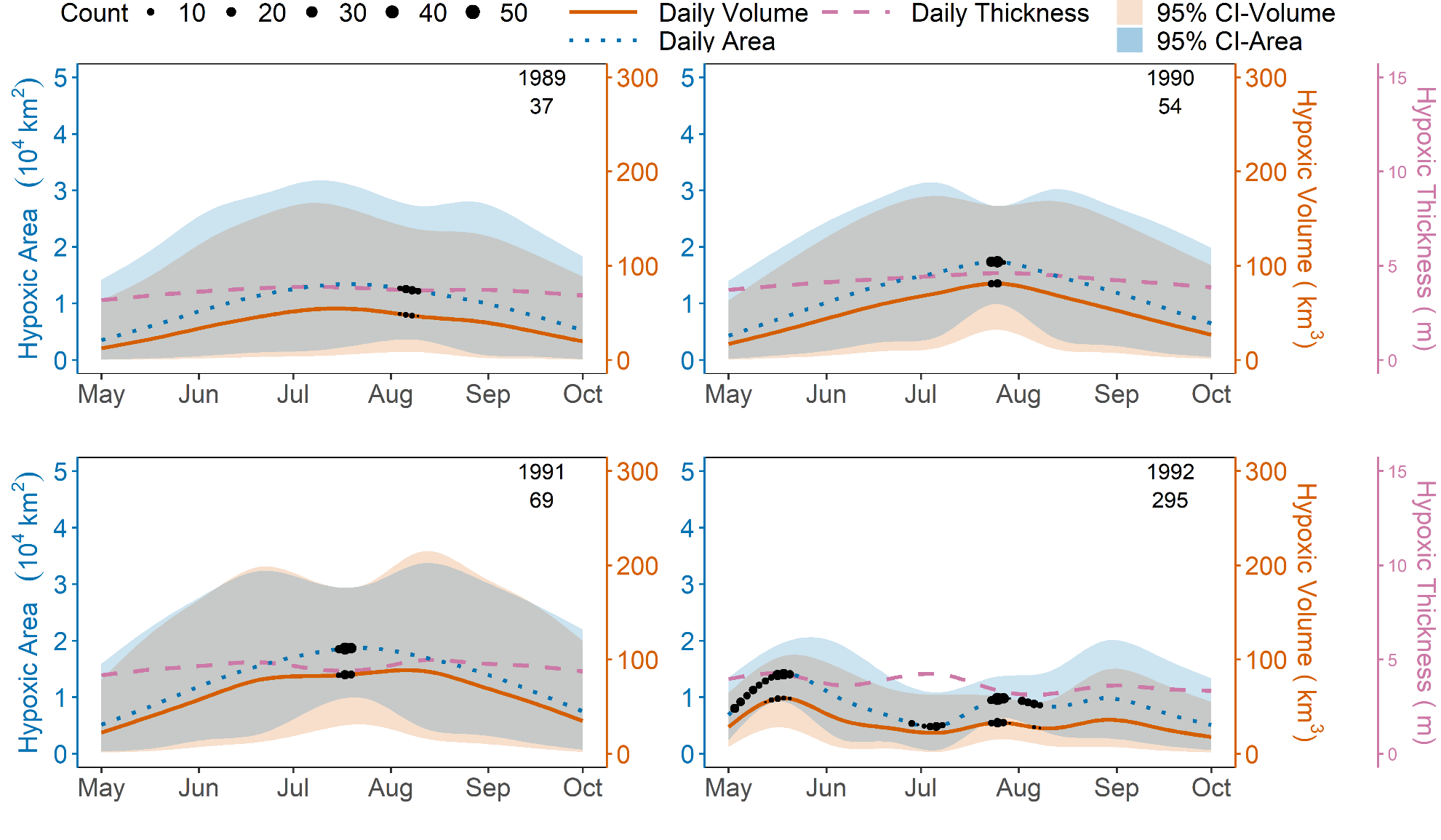


Figure 6. Summer-wide daily estimates of area, volume, and thickness with the 95% CI of area, and volume for 1989-1992


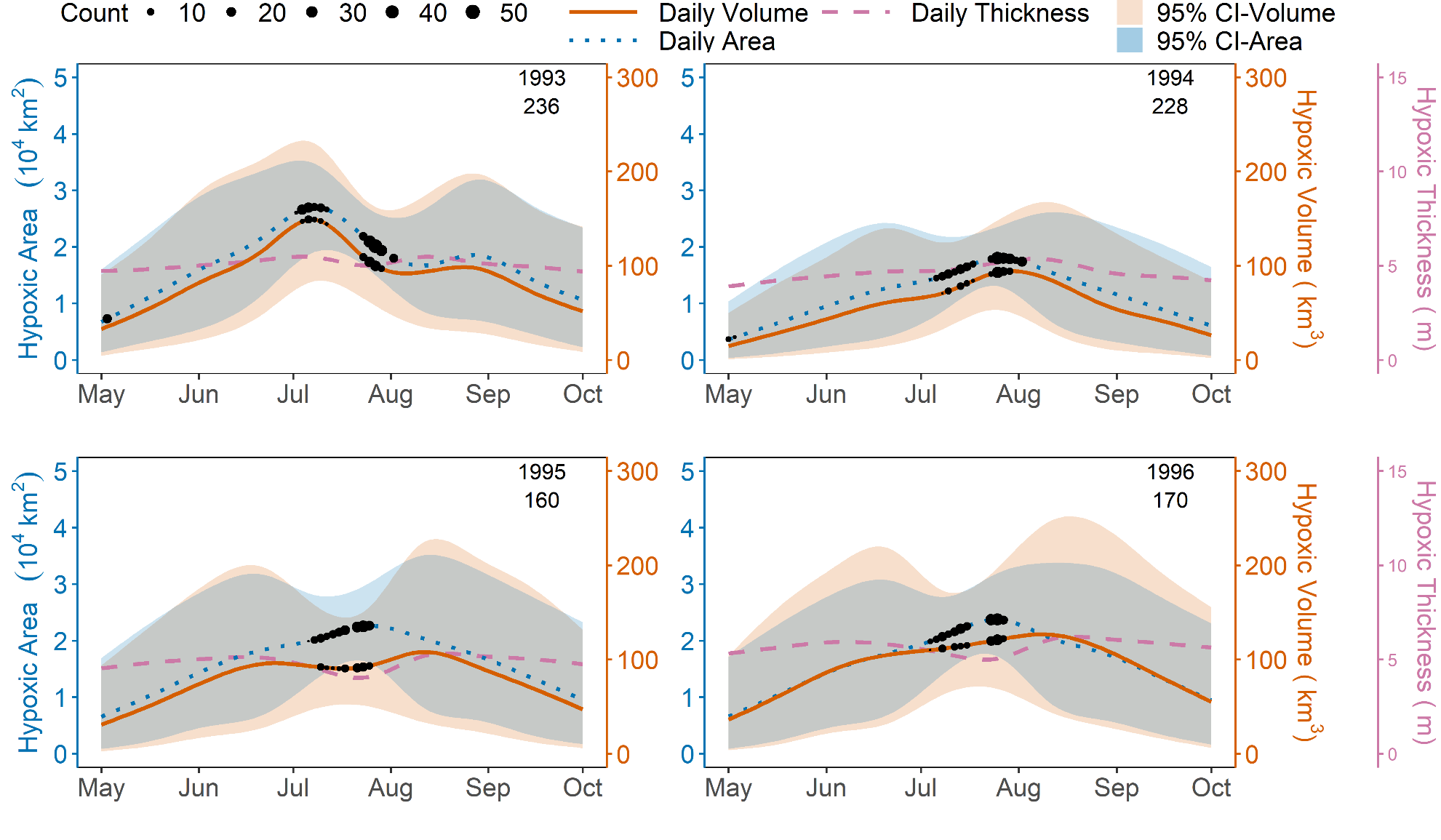


Figure 7. Summer-wide daily estimates of area, volume, and thickness with the 95% CI of area, and volume for 1993-1996


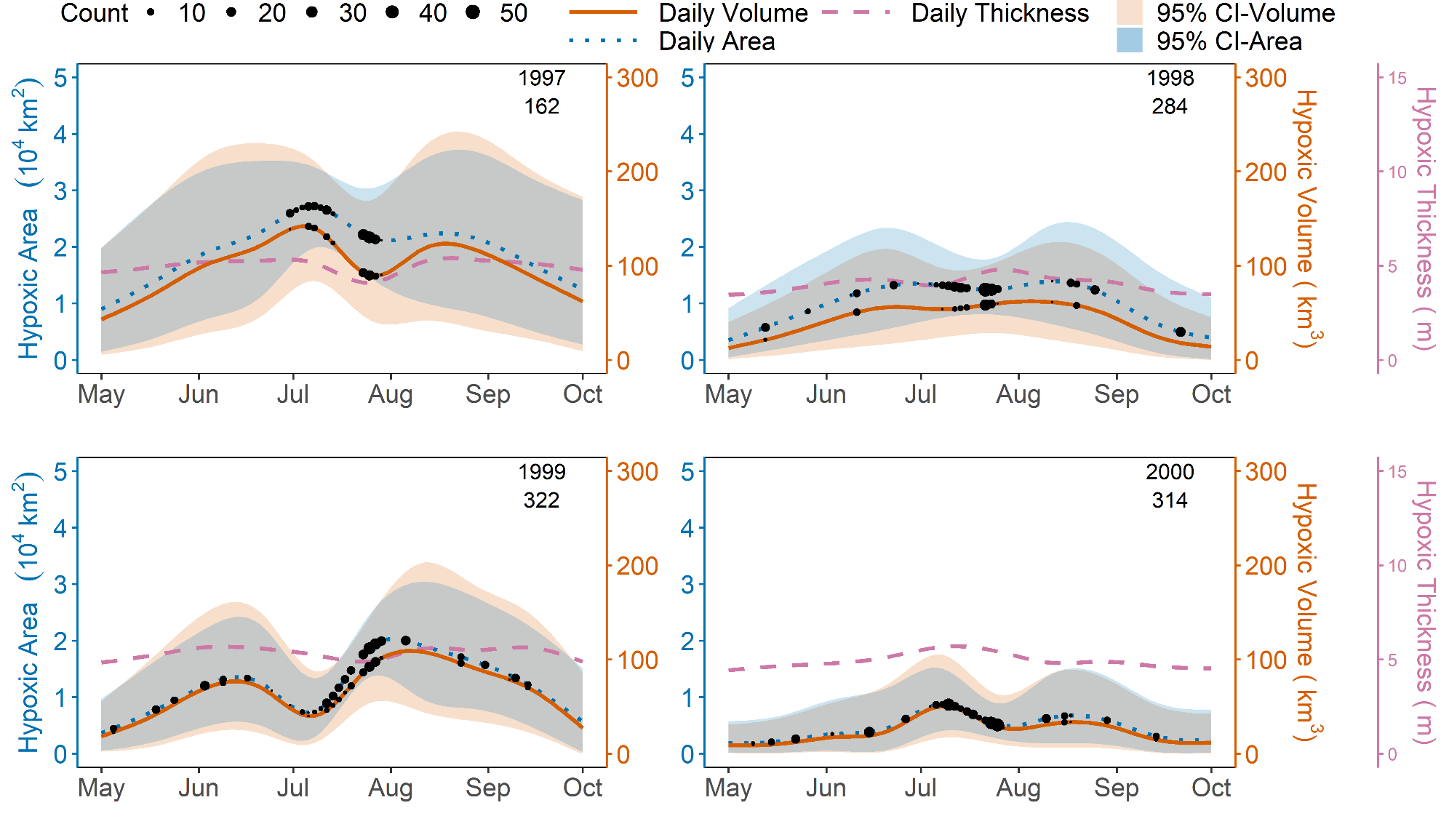


Figure 8. Summer-wide daily estimates of area, volume, and thickness with the 95% CI of area, and volume for 1997-2000


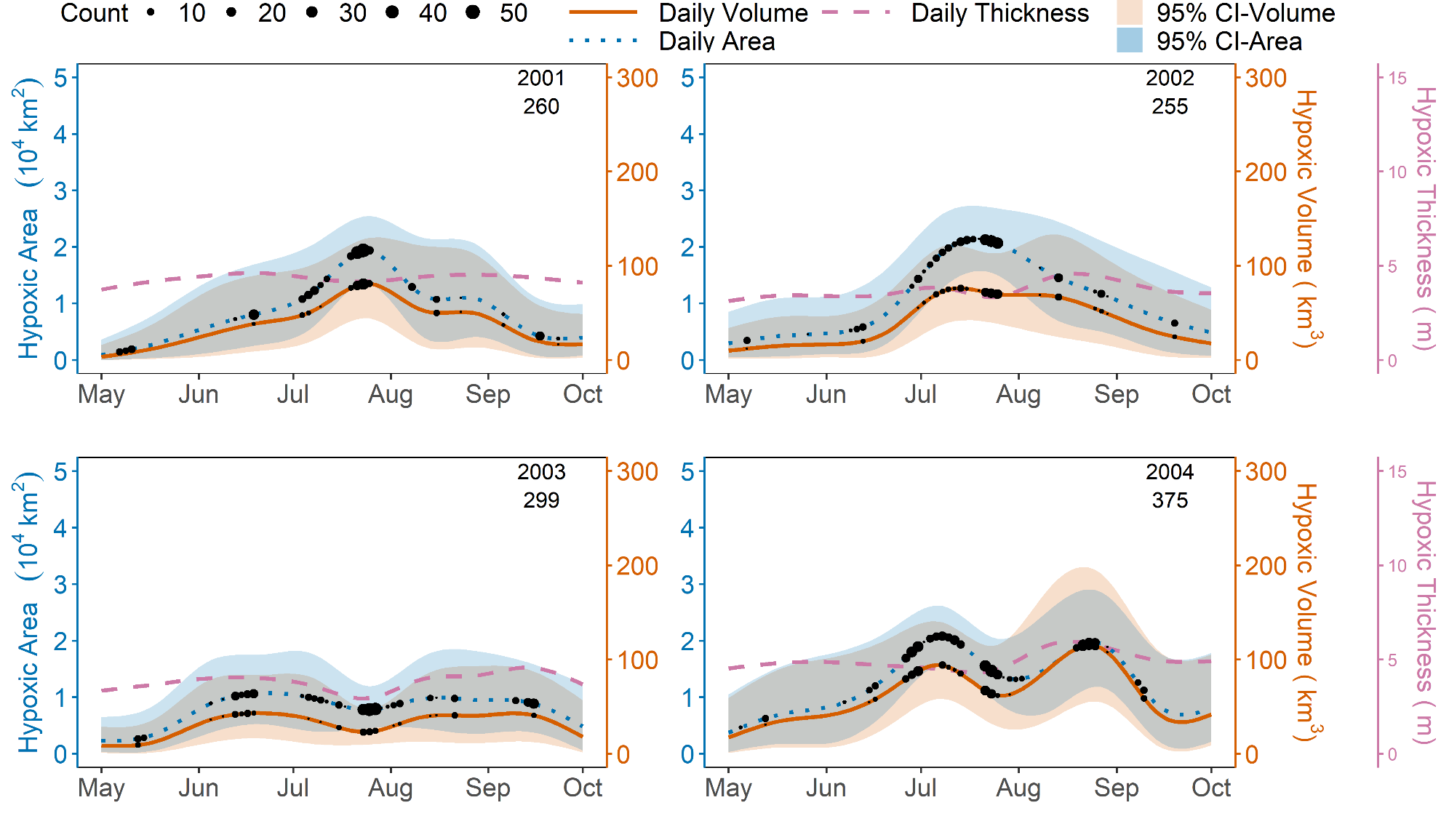


Figure 9. Summer-wide daily estimates of area, volume, and thickness with the 95% CI of area, and volume for 2001-2004


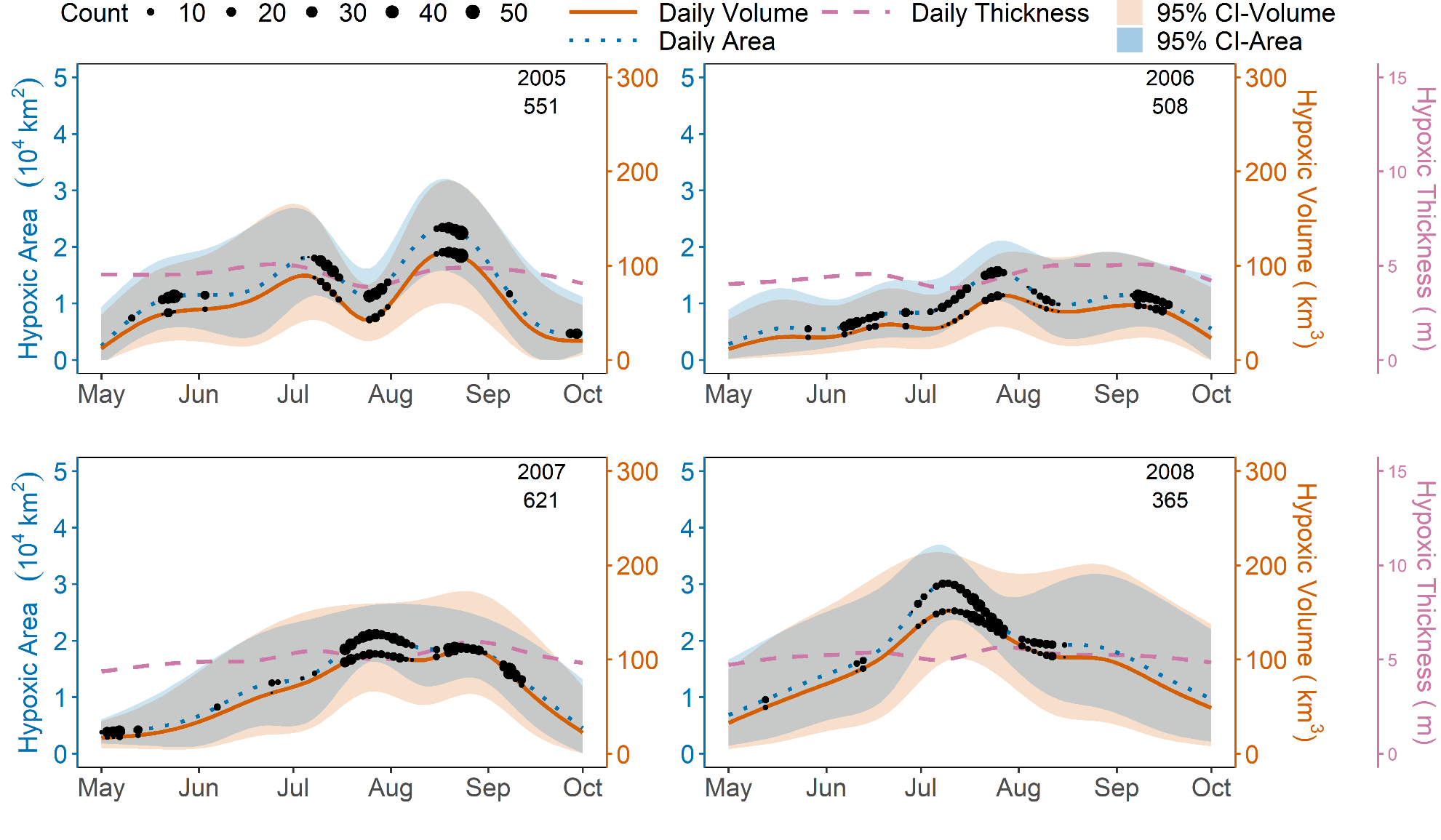


Figure 10. Summer-wide daily estimates of area, volume, and thickness with the 95% CI of area, and volume for 2005-2008


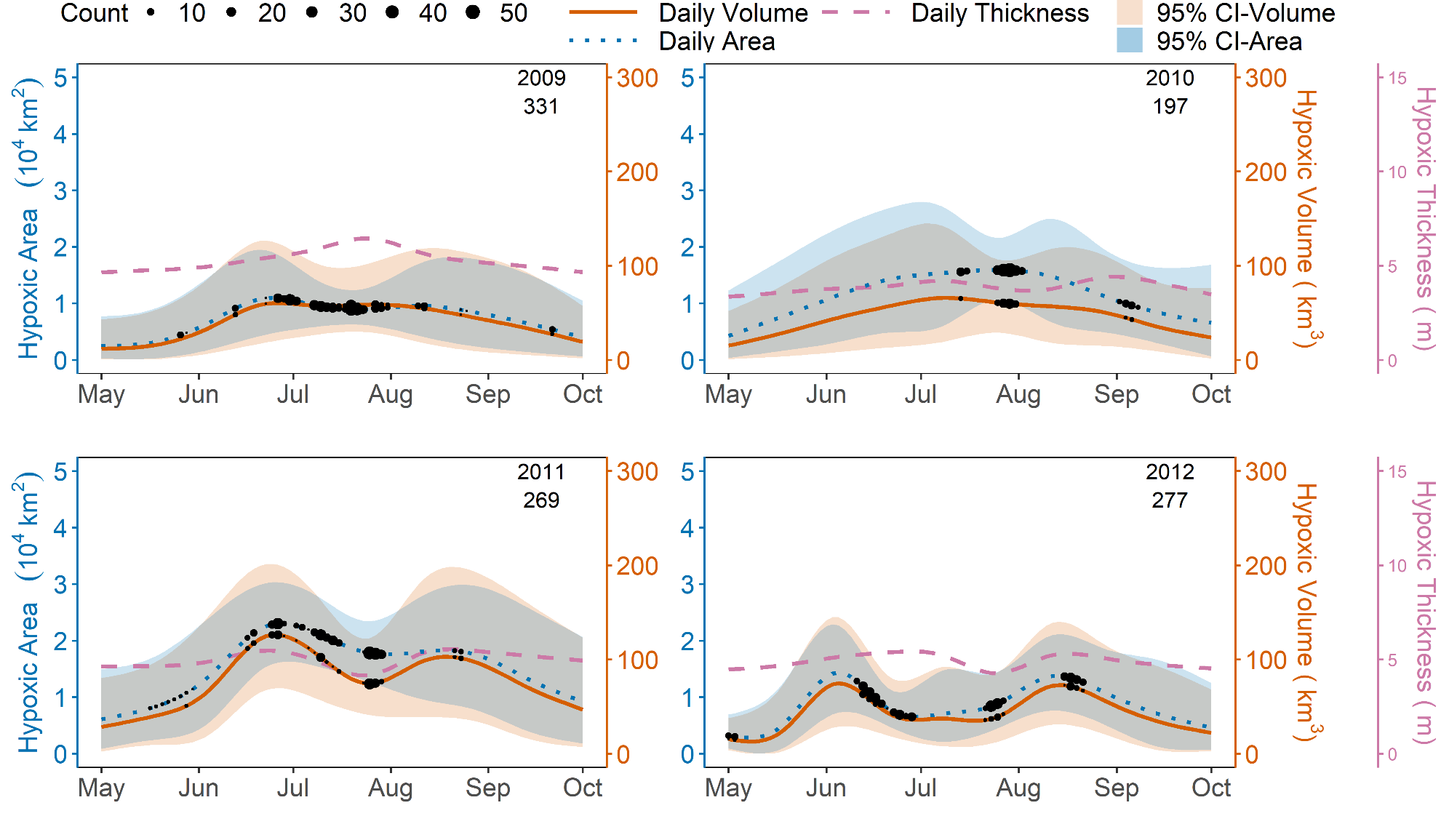


Figure 11. Summer-wide daily estimates of area, volume, and thickness with the 95% CI of area, and volume for 2009-2012


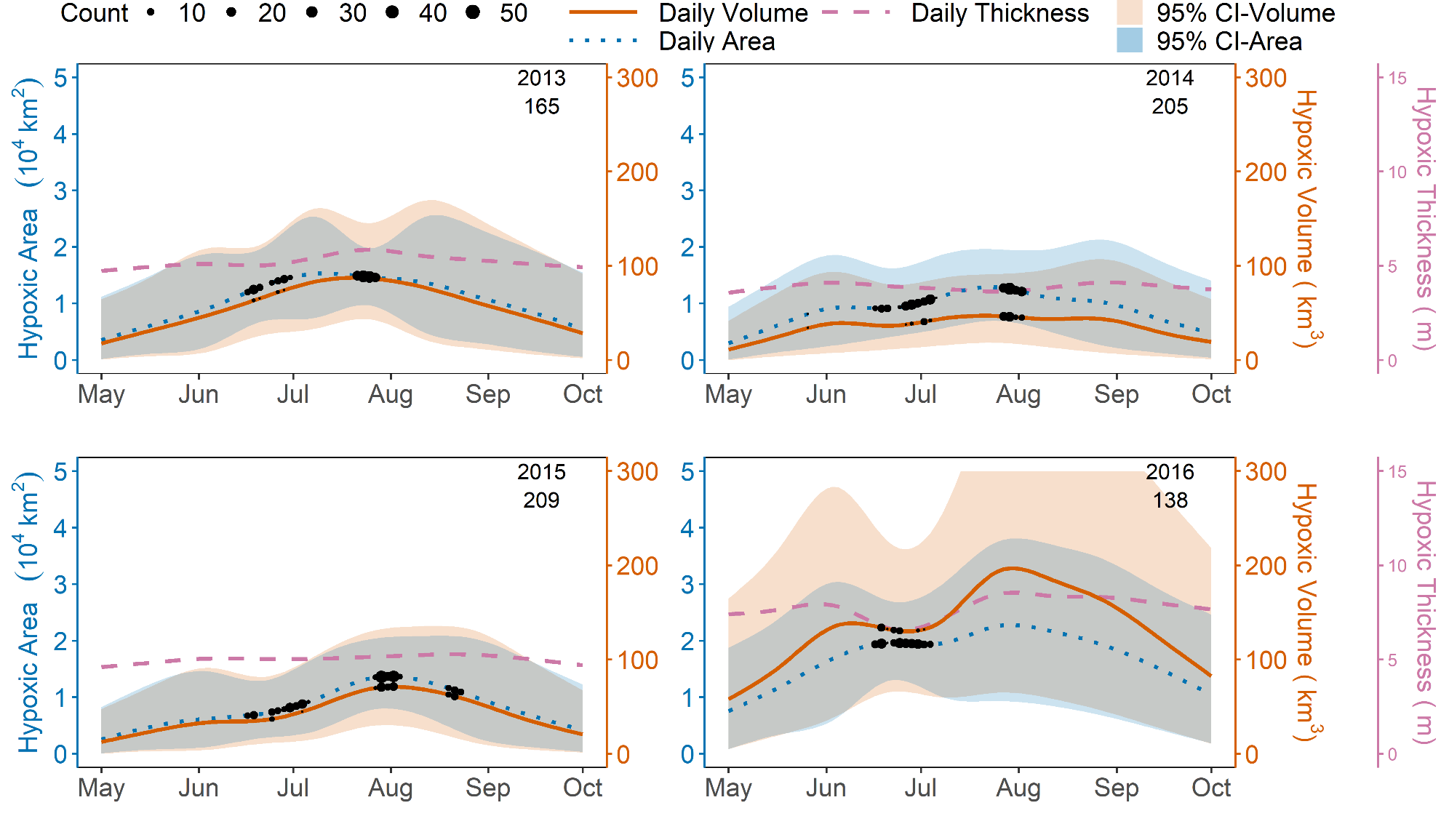


Figure 12. Summer-wide daily estimates of area, volume, and thickness with the 95% CI of area, and volume for 2013-2016


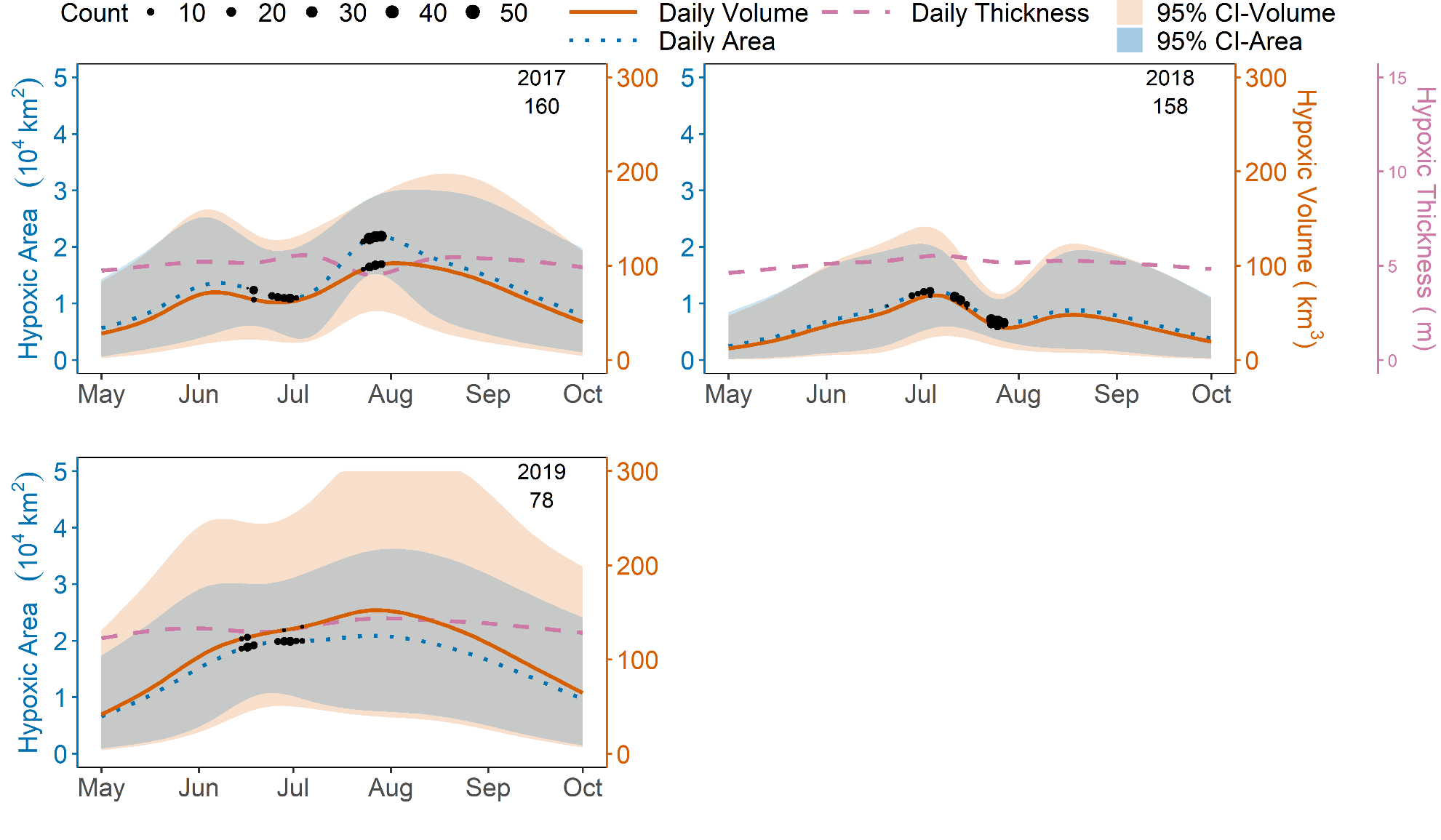


Figure 13. Summer-wide daily estimates of area, volume, and thickness with the 95% CI of area, and volume for 2017-2019

# **Summary of histogram data from model verification**

Table 4. Count of observations and simulations from model with and without INT

|  | Count | | Percentage |
| --- | --- | --- | --- |
|  | HT>0 | Total | HT>0 |
| Observations | 2564 | 6731 | 38% |
| Model with INT | 157371865 | 786870000 | 20% |
| Model without INT | 103866840 | 786870000 | 15% |

Table 5. Mean thickness from various sections and shelfwide for observations and models with and without INT

| Section | Thickness (m) | | |
| --- | --- | --- | --- |
|  | Observations | Model with INT | Model without INT |
| East - Shallow | 3.74 | 3.20 | 2.29 |
| East - Deep | 5.34 | 8.10 | 5.48 |
| West - Shallow | 3.05 | 2.62 | 2.62 |
| West - Deep | 4.32 | 6.48 | 4.31 |
| Shelfwide (HT>0) | 2.99 | 4.87 | 3.17 |
| Shelfwide (all) | 1.48 | 1.03 | 0.57 |

# **Comparison of model results**

Our MC estimates can be compared to those of Obenour et al., (2013), which did not include the INT. For the common period (1985-2011), correlations (r^2^) of our new volume and thickness estimates with the previous MC estimates are 0.85 and 0.76, respectively. The hypoxic area correlation is much higher (0.96) since the INT only affects thickness and volume estimation. On average, our new MC estimates of volume are 11% higher than the results from Obenour et al., (2013), but differences in estimates from individual years range from -21% to 81%. The highest change in volume (81%) was for 2000, which was a year of unusually mild hypoxia due to drought, so this increase translated to an absolute change of only 12 km^3^. The years most likely to have maximum and minimum MC hypoxic volumes (2008 and 1988, respectively) remain unchanged. However, the confidence intervals in volume estimates from the current model are 36% wider compared to the MC estimates from Obenour et al., (2013), comparable to a 30% increase in uncertainty in area estimates from Matli et al., (2018). The model structure used in Obenour et al., (2013) did not include the temporal aspects of the observations collected in a given cruise, which is similar to assuming that all observations in a cruise are collected at the same time. Our space-time model considers shelfwide cruises more realistically as multiday events. Because not all samples are collected on the estimation date (the middle of the cruise period), temporal stochasticity adds to the estimation uncertainty.

References

1. Obenour, D. R., Scavia, D., Rabalais, N. N., Turner, R. E., & Michalak, A. M. (2013). Retrospective analysis of midsummer hypoxic area and volume in the northern Gulf of Mexico, 1985–2011. *Environmental Science & Technology*, *47*(17), 9808-9815.

2. Matli, V. R. R., Fang, S., Guinness, J., Rabalais, N. N., Craig, J. K., & Obenour, D. R. (2018). Space-time geostatistical assessment of hypoxia in the northern Gulf of Mexico. *Environmental Science & Technology*, *52*(21), 12484-12493.

# **Summary of area, volume, and thickness estimates of hypoxia**


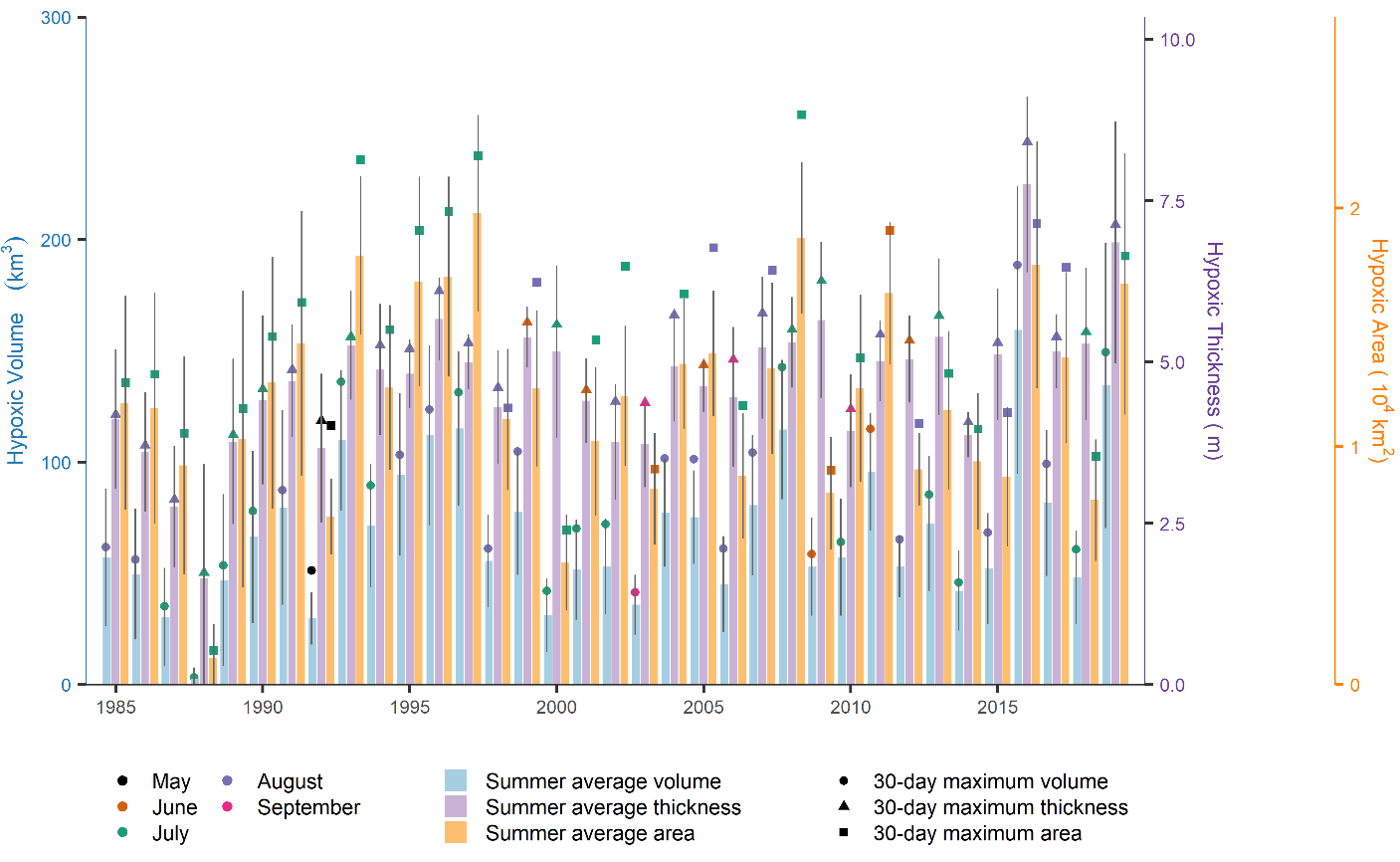

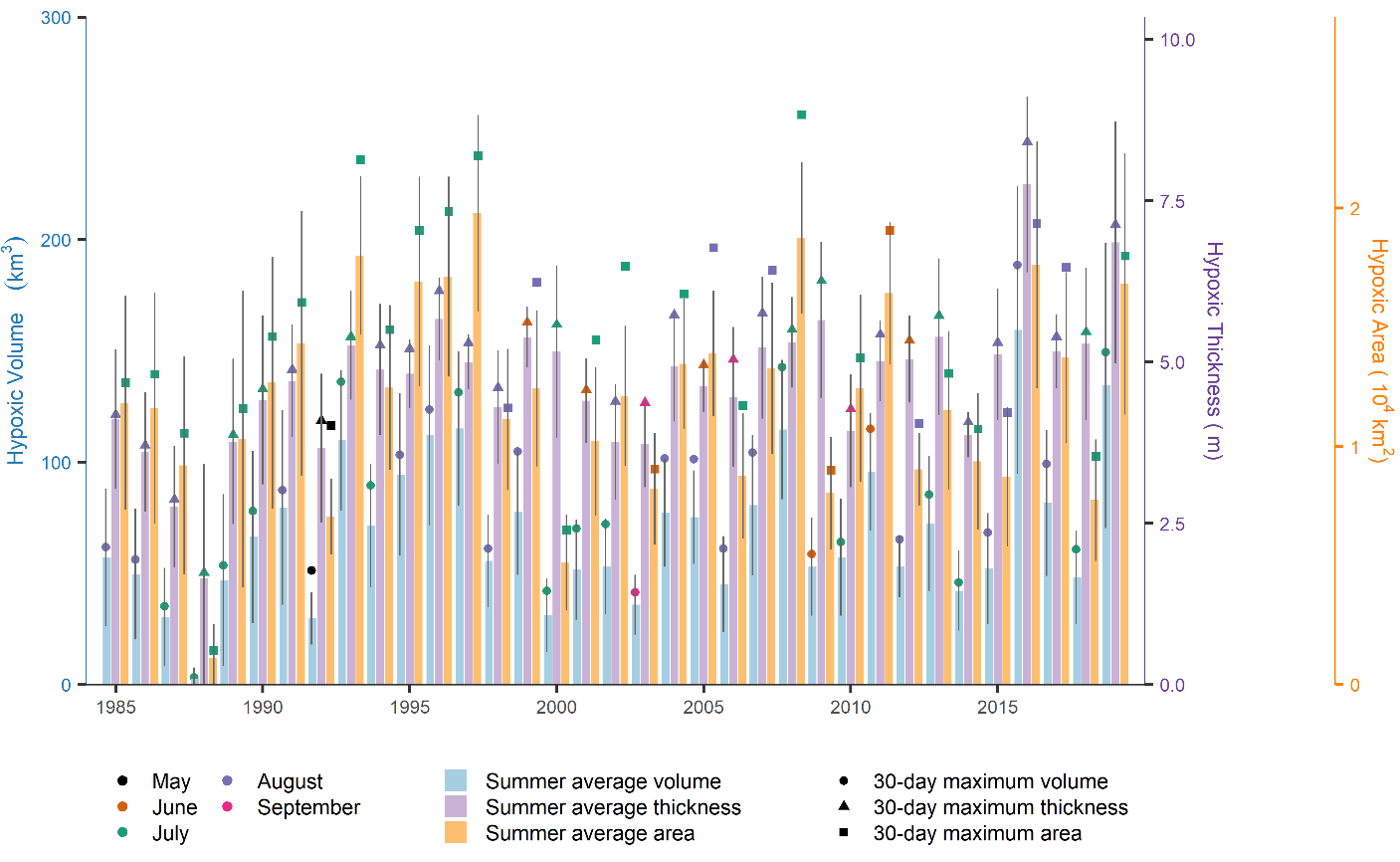


Fig 14. Summerwide average (June-August) and 30-day maximum estimates of hypoxic area, volume, and thickness. The results are color-coded by the month in which daily maximum is observed.

# **Summary of predictive regressions for hypoxia metrics.**

Table 6. Summary of regression analyses and coefficients for BIC-selected variables for various hypoxic metrics across various regions of our study area.

| *Metric* | *Region* | *Period* | *R^2^* | *Intercept* | *year* | *spring-summer N loads*  *(Gg/mo)* | *summer westerlies*  *(m/s)* | *spring-summer westerlies*  *(m/s)* | *winter N loads*  *(Gg/mo)* | *weighted windspeed squared*  *(m^2^/S3)* | *summer flows*  *(m^3^/s)* | *Interaction between spring-summer N loads and westerlies*  *(Gg-m/mo-s)* |
| --- | --- | --- | --- | --- | --- | --- | --- | --- | --- | --- | --- | --- |
| **A** | *SW* | *MC* | *0.48* | -0.65 |  | 0.087 |  | -3.42 |  |  |  |  |
|  |  | *SA* | *0.38* | 1.25 |  | 0.062 |  |  | 0.07 |  |  |  |
|  | *E* | *MC* | *0.56* | 2.53 |  | 0.041 | 0.57 |  |  | -0.07 |  |  |
|  |  | *SA* | *0.37* | 1.14 |  |  |  |  | 0.03 |  | 0.12 |  |
|  | *W* | *MC* | *0.48* | -0.50 |  | 0.207 | -2.01 | -3.32 |  |  |  |  |
|  |  | *SA* | *0.48* | -1.77 |  | 0.211 | -3.13 |  | 0.25 |  |  |  |
| **V** | *SW* | *MC* | *0.36* | -14.87 |  | 0.619 |  |  |  |  |  |  |
|  |  | *SA* | *0.28* | 10.65 |  | 0.482 |  |  |  |  |  |  |
|  | *E* | *MC* | *0.55* | -18.65 |  | 0.579 | 3.33 | -14.63 |  | -0.34 |  | 0.19 |
|  |  | *SA* | *0.24* | 6.87 |  | 0.213 |  |  |  |  |  |  |
|  | *W* | *MC* | *0.35* | -11.07 |  | 1.032 | -6.78 | -15.89 |  |  |  |  |
|  |  | *SA* | *0.24* | 7.23 |  | 1.162 |  |  |  |  |  |  |
| **T** | *SW* | *MC* | *0.28* | -78.31 | 0.04 | 0.013 | 0.29 |  |  |  |  |  |
|  |  | *SA* | *0.10* | 3.94 |  | 0.007 |  |  |  |  |  |  |
|  | *E* | *MC* | *0.21* | 4.13 |  |  |  |  |  | -0.04 | 0.10 |  |
|  |  | *SA* | *0.10* | 4.44 |  | 0.011 |  |  |  |  |  |  |
|  | *W* | *MC* | *0.14* | -95.47 | 0.05 |  |  | -0.60 |  |  |  |  |
|  |  | *SA* | *0.07* | 3.69 |  | 0.022 |  |  |  |  |  |  |

Table 7. Response variables used in summer average regressions (SW indicates shelfwide).

| year | Thickness – SW (m) | Area – SW (km^2^) | Volume – SW (km^3^) | Thickness – East (m) | Area – East (km^2^) | Volume – East (km^3^) | Thickness – West (m) | Area – West (km^2^) | Volume - West (km^3^) |
| --- | --- | --- | --- | --- | --- | --- | --- | --- | --- |
| 1992 | 3.67 | 8070 | 29.8 | 4.06 | 3849 | 16.3 | 3.18 | 4220 | 13.5 |
| 1993 | 5.26 | 20638 | 109.8 | 5.93 | 6617 | 40.3 | 4.83 | 14021 | 69.5 |
| 1994 | 4.88 | 14287 | 71.4 | 5.04 | 4725 | 24.5 | 4.74 | 9562 | 46.9 |
| 1995 | 4.81 | 19388 | 94.3 | 5.72 | 5589 | 33.0 | 4.37 | 13799 | 61.3 |
| 1996 | 5.67 | 19618 | 112.1 | 6.62 | 6007 | 40.6 | 5.18 | 13610 | 71.5 |
| 1997 | 5.00 | 22673 | 115.1 | 5.73 | 6613 | 39.4 | 4.62 | 16060 | 75.7 |
| 1998 | 4.30 | 12745 | 55.5 | 5.07 | 5590 | 29.3 | 3.54 | 7156 | 26.2 |
| 1999 | 5.39 | 14251 | 77.6 | 6.29 | 5071 | 32.8 | 4.76 | 9180 | 44.8 |
| 2000 | 5.16 | 5853 | 31.2 | 5.47 | 1979 | 11.1 | 5.01 | 3874 | 20.1 |
| 2001 | 4.40 | 11695 | 51.6 | 4.61 | 3457 | 16.3 | 4.19 | 8239 | 35.4 |
| 2002 | 3.76 | 13879 | 53.1 | 4.24 | 4072 | 18.4 | 3.48 | 9807 | 34.8 |
| 2003 | 3.73 | 9424 | 35.8 | 4.21 | 3783 | 16.7 | 3.30 | 5641 | 19.1 |
| 2004 | 4.93 | 15417 | 77.2 | 5.89 | 4590 | 27.9 | 4.46 | 10827 | 49.2 |
| 2005 | 4.62 | 15922 | 75.3 | 5.29 | 3964 | 22.2 | 4.34 | 11958 | 53.0 |
| 2006 | 4.46 | 10037 | 45.2 | 5.22 | 3543 | 19.0 | 3.90 | 6493 | 26.2 |
| 2007 | 5.22 | 15210 | 80.6 | 5.93 | 4167 | 25.6 | 4.90 | 11043 | 55.0 |
| 2008 | 5.30 | 21483 | 114.6 | 6.00 | 6162 | 38.0 | 4.96 | 15320 | 76.6 |
| 2009 | 5.65 | 9201 | 52.9 | 6.09 | 4400 | 28.3 | 5.04 | 4800 | 24.7 |
| 2010 | 3.93 | 14258 | 57.2 | 4.42 | 4149 | 19.4 | 3.65 | 10110 | 37.8 |
| 2011 | 5.02 | 18824 | 95.7 | 6.05 | 6126 | 38.3 | 4.44 | 12698 | 57.4 |
| 2012 | 5.04 | 10357 | 53.1 | 5.88 | 4007 | 24.5 | 4.42 | 6350 | 28.6 |
| 2013 | 5.39 | 13190 | 72.3 | 5.79 | 3984 | 24.1 | 5.06 | 9206 | 48.2 |
| 2014 | 3.87 | 10734 | 42.2 | 4.31 | 4063 | 18.2 | 3.49 | 6671 | 23.9 |
| 2015 | 5.12 | 10000 | 52.1 | 5.79 | 3891 | 23.2 | 4.59 | 6109 | 28.9 |

Table 8. Candidate variables used in Summer Average regressions (MR indicates Mississippi River, AR indicates Atchafalaya River, W indicates western shelf).

| year | Spring N loads – AR (Gg/mo) | Spring N loads – MR (Gg/mo) | Spring N loads - MR+AR (Gg/mo) | Summer Westerlies – W (m/s) | Winter N loads - MR+AR (Gg/mo) | Winter N loads – MR (Gg/mo) | Winter N loads – AR (Gg/mo) | Summer flows – MR (m^3^/s) |
| --- | --- | --- | --- | --- | --- | --- | --- | --- |
| 1992 | 17.16 | 72.39 | 89.55 | 0.18 | 95.64 | 73.82 | 21.82 | 12.15 |
| 1993 | 49.47 | 138.42 | 187.89 | -0.10 | 129.12 | 100.58 | 28.54 | 21.10 |
| 1994 | 27.10 | 69.11 | 96.21 | -0.43 | 92.14 | 69.61 | 22.53 | 10.39 |
| 1995 | 33.03 | 93.97 | 127.00 | -0.39 | 63.96 | 48.77 | 15.19 | 19.89 |
| 1996 | 33.16 | 93.64 | 126.80 | -0.58 | 52.08 | 39.77 | 12.31 | 17.49 |
| 1997 | 31.99 | 91.57 | 123.56 | 0.15 | 106.50 | 80.45 | 26.05 | 14.86 |
| 1998 | 41.76 | 108.91 | 150.67 | 0.44 | 80.30 | 61.01 | 19.29 | 15.75 |
| 1999 | 37.23 | 110.31 | 147.54 | -0.53 | 96.55 | 75.22 | 21.32 | 12.87 |
| 2000 | 22.86 | 63.14 | 86.01 | 0.01 | 32.95 | 25.04 | 7.92 | 11.10 |
| 2001 | 26.35 | 101.55 | 127.90 | -0.49 | 73.53 | 58.21 | 15.32 | 12.63 |
| 2002 | 29.60 | 91.71 | 121.32 | -1.22 | 63.08 | 50.28 | 12.80 | 13.26 |
| 2003 | 23.43 | 64.61 | 88.03 | -0.29 | 52.57 | 40.37 | 12.21 | 14.26 |
| 2004 | 28.44 | 84.52 | 112.96 | -0.57 | 70.81 | 55.24 | 15.57 | 16.32 |
| 2005 | 22.67 | 58.17 | 80.84 | -0.51 | 103.39 | 79.73 | 23.66 | 7.96 |
| 2006 | 21.01 | 58.69 | 79.70 | -0.22 | 51.21 | 38.35 | 12.86 | 6.67 |
| 2007 | 24.32 | 89.78 | 114.10 | -0.38 | 78.37 | 59.23 | 19.15 | 11.16 |
| 2008 | 39.87 | 136.71 | 176.59 | 0.39 | 90.36 | 68.32 | 22.04 | 18.13 |
| 2009 | 33.04 | 100.02 | 133.05 | 0.82 | 76.59 | 56.02 | 20.57 | 16.66 |
| 2010 | 32.16 | 93.21 | 125.37 | -0.26 | 105.40 | 79.49 | 25.91 | 16.49 |
| 2011 | 48.63 | 113.72 | 162.34 | 0.70 | 71.50 | 50.33 | 21.17 | 19.20 |
| 2012 | 15.01 | 37.12 | 52.13 | 0.30 | 69.03 | 48.71 | 20.32 | 5.24 |
| 2013 | 38.82 | 110.14 | 148.96 | 0.22 | 56.24 | 40.20 | 16.05 | 17.11 |
| 2014 | 28.32 | 77.97 | 106.29 | 0.06 | 52.54 | 38.77 | 13.77 | 12.96 |
| 2015 | 30.13 | 107.24 | 137.37 | 0.54 | 53.99 | 42.12 | 11.87 | 23.40 |

Table 9. Response variables used in Midsummer Cruise regressions (SW indicates shelfwide)

| year | Thickness – SW (m) | Area – SW (km^2^) | Volume – SW (km^3^) | Thickness – East (m) | Area – East (km^2^) | Volume – East (km^3^) | Thickness – West (m) | Area – West (km^2^) | Volume - West (km^3^) |
| --- | --- | --- | --- | --- | --- | --- | --- | --- | --- |
| 1985 | 3.82 | 14095 | 54.3 | 4.71 | 6103 | 29.0 | 3.10 | 7993 | 25.3 |
| 1986 | 3.41 | 13842 | 48.1 | 4.17 | 3936 | 17.0 | 3.05 | 9906 | 31.1 |
| 1987 | 2.52 | 11858 | 30.8 | 2.92 | 1895 | 5.8 | 2.42 | 9963 | 24.9 |
| 1988 | 1.61 | 862 | 1.4 | 1.59 | 377 | 0.7 | 1.45 | 485 | 0.8 |
| 1989 | 3.52 | 12670 | 45.9 | 3.56 | 4283 | 15.5 | 3.43 | 8388 | 30.4 |
| 1990 | 4.64 | 17593 | 82.3 | 5.22 | 6472 | 34.1 | 4.25 | 11121 | 48.2 |
| 1991 | 4.09 | 19012 | 79.6 | 4.15 | 5131 | 21.8 | 4.03 | 13881 | 57.8 |
| 1992 | 3.39 | 10233 | 35.0 | 3.54 | 5460 | 19.5 | 3.17 | 4773 | 15.5 |
| 1993 | 4.75 | 20782 | 98.9 | 5.81 | 8171 | 47.8 | 4.05 | 12611 | 51.1 |
| 1994 | 5.14 | 18540 | 95.8 | 4.15 | 6699 | 28.1 | 5.69 | 11841 | 67.7 |
| 1995 | 3.78 | 23081 | 88.2 | 4.40 | 6472 | 28.6 | 3.50 | 16609 | 59.5 |
| 1996 | 4.75 | 24019 | 115.2 | 5.10 | 7683 | 39.4 | 4.55 | 16336 | 75.9 |
| 1997 | 3.86 | 21872 | 85.9 | 3.56 | 5502 | 19.9 | 3.93 | 16370 | 66.0 |
| 1998 | 4.93 | 13273 | 65.5 | 6.09 | 7573 | 46.3 | 3.23 | 5701 | 19.2 |
| 1999 | 4.73 | 18420 | 87.5 | 5.55 | 5704 | 31.9 | 4.33 | 12716 | 55.6 |
| 2000 | 5.35 | 4958 | 27.2 | 4.83 | 2951 | 14.6 | 5.99 | 2007 | 12.6 |
| 2001 | 3.97 | 19674 | 78.4 | 3.23 | 5074 | 16.5 | 4.21 | 14600 | 61.9 |
| 2002 | 3.27 | 21870 | 71.9 | 3.35 | 5230 | 17.8 | 3.23 | 16640 | 54.2 |
| 2003 | 2.82 | 6733 | 19.2 | 2.86 | 2209 | 6.4 | 2.77 | 4524 | 12.8 |
| 2004 | 4.18 | 15514 | 65.7 | 4.91 | 5067 | 25.4 | 3.76 | 10446 | 40.3 |
| 2005 | 3.55 | 11469 | 41.3 | 3.59 | 2348 | 8.7 | 3.51 | 9121 | 32.6 |
| 2006 | 4.18 | 15928 | 67.0 | 4.56 | 5074 | 23.4 | 3.96 | 10854 | 43.6 |
| 2007 | 4.77 | 20220 | 97.0 | 5.16 | 4413 | 23.1 | 4.64 | 15807 | 73.9 |
| 2008 | 5.55 | 23134 | 128.9 | 5.74 | 6938 | 40.4 | 5.44 | 16197 | 88.5 |
| 2009 | 6.07 | 8233 | 50.4 | 6.26 | 6031 | 38.0 | 5.18 | 2201 | 12.4 |
| 2010 | 3.66 | 16398 | 60.5 | 4.32 | 3675 | 16.6 | 3.43 | 12724 | 44.0 |
| 2011 | 4.07 | 17937 | 73.4 | 4.86 | 6490 | 31.8 | 3.58 | 11447 | 41.6 |
| 2012 | 4.08 | 7967 | 33.3 | 5.52 | 1636 | 9.7 | 3.60 | 6331 | 23.6 |
| 2013 | 6.07 | 15003 | 91.3 | 4.59 | 3185 | 14.8 | 6.46 | 11818 | 76.5 |
| 2014 | 3.31 | 12504 | 41.7 | 4.01 | 5828 | 23.5 | 2.65 | 6676 | 18.2 |
| 2015 | 5.06 | 13486 | 69.2 | 5.74 | 5169 | 30.4 | 4.58 | 8317 | 38.8 |

Table 10. Candidate variables used in Midsummer Cruise regressions (MR indicates Mississippi River, AR indicates Atchafalaya River, E indicates eastern shelf, W indicates western shelf, SW indicates shelfwide)

| year | Spring N loads – AR (Gg/mo) | Spring N loads – MR (Gg/mo) | Spring N loads - MR+AR (Gg/mo) | Summer Westerlies – E (m/s) | Summer Westerlies – W (m/s) | Summer Westerlies – SW (m/s) | Spring Westerlies – E (m/s) | Spring Westerlies – W (m/s) | Spring Westerlies – SW (m/s) | Weighted windspeed squared – E (m^2^/S3) | Summer flows – MR (m^3^/s) |
| --- | --- | --- | --- | --- | --- | --- | --- | --- | --- | --- | --- |
| 1985 | 30.16 | 98.03 | 128.19 | 0.36 | -0.05 | 0.15 | -0.70 | -0.67 | -0.68 | 18.32 | 9.26 |
| 1986 | 31.68 | 107.21 | 138.89 | -0.05 | 0.53 | 0.24 | -1.91 | -1.35 | -1.63 | 20.91 | 11.82 |
| 1987 | 24.45 | 84.63 | 109.08 | -1.58 | -0.76 | -1.17 | -0.34 | -0.33 | -0.34 | 26.31 | 9.36 |
| 1988 | 13.71 | 54.04 | 67.75 | 0.74 | 0.30 | 0.52 | -0.96 | -1.10 | -1.03 | 62.36 | 3.60 |
| 1989 | 18.98 | 76.98 | 95.96 | 0.13 | -0.35 | -0.11 | -1.55 | -1.15 | -1.35 | 34.21 | 19.85 |
| 1990 | 31.69 | 134.15 | 165.83 | -0.53 | -0.85 | -0.69 | -2.12 | -2.01 | -2.06 | 26.80 | 16.05 |
| 1991 | 38.18 | 141.12 | 179.30 | 1.66 | 0.39 | 1.02 | -2.96 | -2.69 | -2.83 | 24.91 | 10.49 |
| 1992 | 18.13 | 81.27 | 99.39 | 0.34 | 0.26 | 0.30 | -0.59 | -0.81 | -0.70 | 17.56 | 11.23 |
| 1993 | 52.55 | 145.98 | 198.53 | 1.03 | 0.82 | 0.93 | -1.85 | -1.55 | -1.70 | 20.89 | 19.30 |
| 1994 | 30.20 | 80.13 | 110.33 | 0.73 | 0.27 | 0.50 | -1.12 | -1.86 | -1.49 | 18.25 | 11.04 |
| 1995 | 33.39 | 100.92 | 134.31 | 1.33 | 0.70 | 1.01 | -1.74 | -2.03 | -1.89 | 29.81 | 17.72 |
| 1996 | 36.48 | 101.27 | 137.75 | 0.29 | -0.13 | 0.08 | -1.42 | -1.74 | -1.58 | 22.49 | 13.84 |
| 1997 | 34.56 | 103.04 | 137.60 | 1.91 | 0.58 | 1.24 | -1.42 | -1.38 | -1.40 | 54.52 | 15.19 |
| 1998 | 41.86 | 114.06 | 155.92 | 1.72 | 0.76 | 1.24 | -0.26 | -0.55 | -0.41 | 29.77 | 19.07 |
| 1999 | 38.32 | 118.22 | 156.54 | -0.13 | -1.11 | -0.62 | -1.27 | -2.15 | -1.71 | 18.02 | 14.56 |
| 2000 | 21.68 | 58.45 | 80.13 | 3.09 | 1.65 | 2.37 | -0.88 | -1.67 | -1.27 | 41.59 | 14.08 |
| 2001 | 28.98 | 114.80 | 143.78 | 0.28 | -0.20 | 0.04 | -2.06 | -2.27 | -2.17 | 26.21 | 11.02 |
| 2002 | 34.83 | 109.35 | 144.18 | -0.65 | -1.14 | -0.90 | -2.19 | -2.44 | -2.31 | 17.18 | 9.70 |
| 2003 | 24.94 | 70.73 | 95.67 | 0.50 | -0.82 | -0.16 | -0.37 | -1.00 | -0.69 | 38.68 | 12.51 |
| 2004 | 28.38 | 85.64 | 114.01 | 0.65 | -0.09 | 0.28 | -1.30 | -1.43 | -1.37 | 26.33 | 17.99 |
| 2005 | 25.20 | 65.68 | 90.88 | -0.67 | -0.58 | -0.62 | -1.34 | -0.83 | -1.09 | 25.60 | 8.31 |
| 2006 | 24.40 | 70.72 | 95.12 | -0.58 | -0.05 | -0.32 | -0.37 | -0.77 | -0.57 | 14.21 | 6.52 |
| 2007 | 26.34 | 104.13 | 130.47 | 0.25 | 0.03 | 0.14 | -2.07 | -1.92 | -2.00 | 18.76 | 13.64 |
| 2008 | 44.36 | 144.29 | 188.66 | -0.37 | -0.31 | -0.34 | -1.32 | -1.10 | -1.21 | 31.65 | 19.82 |
| 2009 | 38.19 | 114.27 | 152.45 | 2.56 | 1.79 | 2.17 | -0.48 | -0.61 | -0.55 | 29.39 | 13.24 |
| 2010 | 35.55 | 98.26 | 133.81 | -2.72 | -1.89 | -2.30 | -2.04 | -1.13 | -1.59 | 32.84 | 16.94 |
| 2011 | 54.32 | 122.97 | 177.29 | 0.84 | 1.00 | 0.92 | -1.32 | -1.23 | -1.28 | 15.96 | 17.27 |
| 2012 | 17.59 | 45.34 | 62.93 | 0.09 | 0.32 | 0.20 | -1.07 | -0.72 | -0.89 | 24.68 | 5.01 |
| 2013 | 40.89 | 118.79 | 159.68 | -0.51 | -0.11 | -0.31 | -1.80 | -0.92 | -1.36 | 36.53 | 17.84 |
| 2014 | 29.21 | 80.31 | 109.53 | 2.07 | 0.84 | 1.46 | -1.59 | -0.65 | -1.12 | 29.62 | 14.17 |
| 2015 | 29.08 | 100.18 | 129.26 | 1.66 | 1.11 | 1.39 | -1.97 | -1.29 | -1.63 | 23.39 | 29.03 |
