# Supplementary material for: Trends and drivers of hypoxic thickness and volume in the northern Gulf of Mexico: 1985–2018
Source: PLoS One. 2024 Dec 5;19(12):e0302759. doi: 10.1371/journal.pone.0302759 (PMC11620451; doi:10.1371/journal.pone.0302759)
Supplement: Attachment — Submitted filename: Response Document.docx [file pone.0302759.s003.docx]

**Response to Reviewer and Journal Comments: *Trends and drivers of hypoxic thickness and volume in the northern Gulf of Mexico: 1985-2018***

Dear Editor and Reviewers:

We are pleased to submit our revised manuscript "Trends and drivers of hypoxic thickness and volume in Northern Gulf of Mexico: 1985-2018". We appreciate the reviewers’ positive and helpful comments. All comments have been addressed and we feel the manuscript has been improved. Our detailed responses to reviewer comments are provided below.

Sincerely

V Rohith Reddy Matli

Note:

Reviewer and journal comments are in *italics*

Responses are in normal text.

**Journal Suggested Corrections:**

*When submitting your revision, we need you to address these additional requirements.*

*1. Please ensure that your manuscript meets PLOS ONE's style requirements, including those for file naming. The PLOS ONE style templates can be found at*

[*https://journals.plos.org/plosone/s/file?id=wjVg/PLOSOne_formatting_sample_main_body.pdf*](https://journals.plos.org/plosone/s/file?id=wjVg/PLOSOne_formatting_sample_main_body.pdf) *and*

[*https://journals.plos.org/plosone/s/file?id=ba62/PLOSOne_formatting_sample_title_authors_affiliations.pdf*](https://journals.plos.org/plosone/s/file?id=ba62/PLOSOne_formatting_sample_title_authors_affiliations.pdf)

We made formatting and styling changes where relevant and needed. This includes adding the figures as separate files and changing to the reference citation formats.

*2. Please note that PLOS ONE has specific guidelines on code sharing for submissions in which author-generated code underpins the findings in the manuscript. In these cases, all author-generated code must be made available without restrictions upon publication of the work. Please review our guidelines at* [*https://journals.plos.org/plosone/s/materials-and-software-sharing#loc-sharing-code*](https://journals.plos.org/plosone/s/materials-and-software-sharing#loc-sharing-code) *and ensure that your code is shared in a way that follows best practice and facilitates reproducibility and reuse.*

All the base data and code files for model development and output processing have been uploaded to a Zenodo archive and attached as a doi with the instructions needed to run the model. (DOI: [https://doi.org/zenodo.13145165](https://doi.org/10.5281/zenodo.13145165))

*3. Thank you for stating the following financial disclosure:*

*“This work was funded by NOAA grant NA16NOS4780203.”*

*Please state what role the funders took in the study.  If the funders had no role, please state: "The funders had no role in study design, data collection and analysis, decision to publish, or preparation of the manuscript."*

*If this statement is not correct you must amend it as needed.*

*Please include this amended Role of Funder statement in your cover letter; we will change the online submission form on your behalf.*

We made relevant changes to the manuscript as suggested and added the role of funder statement to the cover letter.

*4. Thank you for stating the following in the Acknowledgments Section of your manuscript:*

*“This work was funded by NOAA grant NA16NOS4780203. This is NGOMEX contribution ###.”*

*We note that you have provided funding information that is currently declared in your Funding Statement. However, funding information should not appear in the Acknowledgments section or other areas of your manuscript. We will only publish funding information present in the Funding Statement section of the online submission form.*

*Please remove any funding-related text from the manuscript and let us know how you would like to update your Funding Statement. Currently, your Funding Statement reads as follows:*

*“This work was funded by NOAA grant NA16NOS4780203.”*

*Please include your amended statements within your cover letter; we will change the online submission form on your behalf.*

We made relevant changes to the manuscript as suggested and removed all the funding information from the submitted manuscript.

*5. When completing the data availability statement of the submission form, you indicated that you will make your data available on acceptance. We strongly recommend all authors decide on a data sharing plan before acceptance, as the process can be lengthy and hold up publication timelines. Please note that, though access restrictions are acceptable now, your entire data will need to be made freely accessible if your manuscript is accepted for publication. This policy applies to all data except where public deposition would breach compliance with the protocol approved by your research ethics board. If you are unable to adhere to our open data policy, please kindly revise your statement to explain your reasoning and we will seek the editor's input on an exemption. Please be assured that, once you have provided your new statement, the assessment of your exemption will not hold up the peer review process.*

We remain committed to sharing all the data from our modelling study and have created a Dryad repository with all the raw simulations. The data has been posted at this DOI (<https://doi.org/10.5061/dryad.q2bvq83tw>), which will be publicly available when linked to the DOI for the accepted manuscript (i.e., once we receive a manuscript DOI, we will be able to publicly activate the Dryad DOI). A reviewer copy is available here (<https://datadryad.org/stash/share/AQktYZbyXudBUESThKFbHjQD7OE86NAbjctOc8u9Ksc>).

The code required to process and summarize these simulations is provided in the Zenodo archive (described in response to comment #2).

*6. We note that Figure 2 in your submission contain [map/satellite] images which may be copyrighted. All PLOS content is published under the Creative Commons Attribution License (CC BY 4.0), which means that the manuscript, images, and Supporting Information files will be freely available online, and any third party is permitted to access, download, copy, distribute, and use these materials in any way, even commercially, with proper attribution. For these reasons, we cannot publish previously copyrighted maps or satellite images created using proprietary data, such as Google software (Google Maps, Street View, and Earth). For more information, see our copyright guidelines:* [*http://journals.plos.org/plosone/s/licenses-and-copyright*](http://journals.plos.org/plosone/s/licenses-and-copyright)*.*

*We require you to either (1) present written permission from the copyright holder to publish these figures specifically under the CC BY 4.0 license, or (2) remove the figures from your submission:*

*1. You may seek permission from the original copyright holder of Figure 2 to publish the content specifically under the CC BY 4.0 license.*

*We recommend that you contact the original copyright holder with the Content Permission Form (*[*http://journals.plos.org/plosone/s/file?id=7c09/content-permission-form.pdf*](http://journals.plos.org/plosone/s/file?id=7c09/content-permission-form.pdf)*) and the following text:*

*“I request permission for the open-access journal PLOS ONE to publish XXX under the Creative Commons Attribution License (CCAL) CC BY 4.0 (*[*http://creativecommons.org/licenses/by/4.0/*](http://creativecommons.org/licenses/by/4.0/)*). Please be aware that this license allows unrestricted use and distribution, even commercially, by third parties. Please reply and provide explicit written permission to publish XXX under a CC BY license and complete the attached form.”*

*Please upload the completed Content Permission Form or other proof of granted permissions as an "Other" file with your submission.*

*In the figure caption of the copyrighted figure, please include the following text: “Reprinted from [ref] under a CC BY license, with permission from [name of publisher], original copyright [original copyright year].”*

*2. If you are unable to obtain permission from the original copyright holder to publish these figures under the CC BY 4.0 license or if the copyright holder’s requirements are incompatible with the CC BY 4.0 license, please either i) remove the figure or ii) supply a replacement figure that complies with the CC BY 4.0 license. Please check copyright information on all replacement figures and update the figure caption with source information. If applicable, please specify in the figure caption text when a figure is similar but not identical to the original image and is therefore for illustrative purposes only.*

*The following resources for replacing copyrighted map figures may be helpful:*

*USGS National Map Viewer (public domain):* [*http://viewer.nationalmap.gov/viewer/*](http://viewer.nationalmap.gov/viewer/)

*The Gateway to Astronaut Photography of Earth (public domain):* [*http://eol.jsc.nasa.gov/sseop/clickmap/*](http://eol.jsc.nasa.gov/sseop/clickmap/)

*Maps at the CIA (public domain):* [*https://www.cia.gov/library/publications/the-world-factbook/index.html*](https://www.cia.gov/library/publications/the-world-factbook/index.html) *and* [*https://www.cia.gov/library/publications/cia-maps-publications/index.html*](https://www.cia.gov/library/publications/cia-maps-publications/index.html)

*NASA Earth Observatory (public domain):* [*http://earthobservatory.nasa.gov/*](http://earthobservatory.nasa.gov/)

*Landsat:* [*http://landsat.visibleearth.nasa.gov/*](http://landsat.visibleearth.nasa.gov/)

*USGS EROS (Earth Resources Observatory and Science (EROS) Center) (public domain):* [*http://eros.usgs.gov/#*](http://eros.usgs.gov/)

*Natural Earth (public domain):* [*http://www.naturalearthdata.com/*](http://www.naturalearthdata.com/)

The basemaps in Figure 2 of our manuscript used material from mapbox designer studio. This material is not copyrighted and mapbox allows free access for usage and sharing with proper attribution. We modified our figures to include this attribution by adding the logo and text as suggested in the guidance of static & print documentation of mapbox designer (<https://docs.mapbox.com/help/dive-deeper/attribution/>).

*7. Please include captions for your Supporting Information files at the end of your manuscript, and update any in-text citations to match accordingly. Please see our Supporting Information guidelines for more information:* [*http://journals.plos.org/plosone/s/supporting-information*](http://journals.plos.org/plosone/s/supporting-information)*.*

We modified the supporting information file to make the modifications as requested and modified our in-text citations to reflect these changes.

**Reviewer Comments:**

*Reviewer #1: The authors derive a geospatial model for predicting hypoxic area, volume and thickness based on real-time data from multiple monitoring programs within the notorious besieged northern Gulf of Mexico hypoxic region. The model utilizes a space-time geostatistical framework and implements a rank-based inverse normal transformation to improve estimates of hypoxic thickness, an important metric typically overlooked. The authors are experts within the arena of geospatial modeling and their geostatistical methodology represents a sound alternative approach. Lack of correlation between hypoxic thickness and the other hypoxic metrics of area and volume, as well as a progressive increasing trend in thickness over the last three decades within the region underscore the importance of hypoxic thickness as an informative metric. The explicit space-time dimensions of the geospatial framework used constitute a novel aspect relative to other approaches, such as biogeochemical process models. Another strength of the model is the validation performed using actual data to identify potential biases precluding prediction accuracy. Potential environmental drivers are also evaluated. Using multiple regression to analyze output from the model, spring nitrogen loading was shown to be the primary driver of hypoxia within the system, in addition to several subsidiary drivers involving meteorological patterns. Subregions within the northern GoM sometimes responded differently to the same meteorological drivers. One of the biggest benefits of the study is the heuristic value in comparison with other approaches aimed at understanding the evolution of hypoxia within the northern Gulf of Mexico.*

Thank you for reviewing our manuscript and considering the major contributions of the study.

*Reviewer #2: PONE reviewer comments Matli & Obenouer
This analysis extends prior analyses of area and volume to include thickness as a metric with which to compare annual and monthly trends and to support resource management decisions. Thickness as a metric is more difficult to understand compared to area (a synthetic number) and volume (also a synthetic number) compared to thickness, which as a synthetic number, is more difficult to understand because it is compiled from multiple hydrographic profiles across a broad range of depths and geographic locations and represents a unified metric that seems less useful as a management tool, either in reduction of nutrient inputs or fishery-related resources.
There is also a mixture of geographically different monitoring cruises, especially differences between TAMU and EPA compared to each other and to LUMCON. Additionally, LUMCONT is a one- or two-day cruise to specific transects contained within the larger LUMCON MC station locations during the shelfwide cruises. It is not clear how these various hydrographic profiles become a monthly or annual metric for statistical analyses.*

Thank you for reviewing our manuscript and providing detailed and helpful comments.

Regarding how the disparate data sources “become a monthly or annual metric”, this is a primary feature of the space-time modeling approach, as highlighted by reviewer #1. The approach allows us to make estimates across time and space considering both spatial and temporal correlation plus large-scale spatial (geographic/bathymetric) and temporal (seasonal trends). We made an edit to the Introduction to help clarify (Line 70-72), but additional details are in the Methods and referenced articles. Even for periods of little data collection, we can still make estimates based mostly on the large-scale trends. However, in cases of extreme data paucity, excessive uncertainty could make the estimates practically useless, as highlighted for 2016 and 2019 at lines 318-324.

*Many stated results, e.g., hypoxia is more likely to occur in July and August than in June, is already well known. Winds from the east in spring disperse nutrients and fresh water from the Mississippi River in the spring season support the phytoplankton biomass that eventually falls to the sea bed and is respired.
Many general terms, e.g., For volume, the corresponding results are 0, 2, 11, 10, and 1 years, respectively, indicating that peak volume tends to lag peak area, at least on average

…..taken together, these results suggest both patterns as well as aberrations in the seasonal….

There is little doubt that a tremendous amount of consistent, strong analytical methods, and a thorough understanding of the metrics provided are correct. These are trademarks of these co-authors.
There needs to be a more concise presentation of the results and their interpretation.*

We reviewed the manuscript and see limited opportunity to make it more concise without cutting out important information. While some results may be “already known” in a general sense, we believe this study provides a more comprehensive and quantitative characterization of hypoxia.

To help address the reviewer’s comment, we have moved the comparison of our new results with those of Obenour et al., 2013 to the supporting information (now referenced at Line 317). We acknowledge that this material will not be particularly important for most readers. In addition, we have also made a few minor edits for clarity and conciseness throughout the manuscript.

*Small things that caught my eye:*

*Inconsistent use of ….hypoxia… as a noun, and …..hypoxic…..as an adjective ……hypoxia metrics, when considered along with other riverine inputs and meteorological factors in multiple regression models. Hypoxic volume,….*

In the literature, we have generally seen “hypoxic” used in front of geometric properties like “area”, “volume”, and “layer”. In most other cases, “hypoxia” is used as an attributive noun, in front of “metrics”, “modeling”, “estimates”. We edited for consistency, in addition to adopting some of your related suggestions (below).

*L36 …..the size of the hypoxic zone*

Corrected at Line 34.

*L 63, Louisiana Universities Marine Consortium*

Corrected at Line 56.

*L 80, thickness of hypoxia across the Gulf hypoxia region from May, second hypoxia should be hypoxic, and so on through the manuscript.*

Corrected at Line 74.

*L 101, change to ….sampling frame….*

Changed at Line 96 and in the supporting information.

Fig. 1, a mixture of font styles

Made changes to the figure to use consistent font throughout the figure and uploaded it as Fig1.tif

*L 172, multiple cruises in each of the years analyzed, but the geographic footprints of those cruises were quite different.*

Made relevant changes to Lines 165-168 to address this comment.

*L 175 et seq. Selection of ‘seasons’ does not appear to be consistent, but rather the months in which there were cruises.*

We use “spring” and “summer” as a shorthand, which is why we put quotes around these terms.

*L186, Mississippi and Atchafalaya rivers*

Changed at Line 181.

*L 212, a lag of 12 days*

Corrected at Line 207.

*L 261, is there a reference for this statement ROMS vs FVCOM?*

The relevant citation is provided in the paragraph’s introductory sentence.

*L 300, …too little variance…*

Existing text appears to be appropriate and consistent with reviewer comment, but we modified it for clarity at Line 293.

*L 336, 2000 was a drought year, little stratification*

Added “due to drought”. This paragraph is now in the supporting information.

*L 354 – 356, these sentences are contradictory. Overall, these results suggest that most of the variability in hypoxic volume can be attributed to changes in hypoxic area. At the same time, variability in hypoxic thickness, which is only modestly correlated to area, is also important.*

We agree these sentences are confusing. They aren’t really necessary, and we have removed them for conciseness.

*L 372-373, is there a conclusion here?*

We think so. It supports the conclusion at Line 442 (end of first paragraph in conclusions).

*Fig. 5, symbols are difficult to see.*

Symbols appear clear in our version. We are submitting high quality figures for publication.

*L 388-389, after reading this paragraph is there any merit to determining hypoxia thickness?*

We think that knowing the hypoxic layer thickness is scientifically relevant. However, it might not be worth predicting. We would prefer not to venture such an opinion in this article.

*L 447, ability to constraint uncertainty…. Change to …constrain…or chloropigments*

Changed at Line 423.

*L 449 “…..Future work in improving this model can focus on incorporating covariates that capture short term changes in system conditions….” Reviewer, of these limitations, it would take dynamic measurements of conductivity, temperature, DO, Chla or chloropigments, possibly turbidity, through the water column with some type of profiler on a buoy that could capture the hypoxic thickness. Or, some type of AUV that can change position, lower raise probes, move to a new position, over some periodicity that would capture short-term variability suitable for the models. OR, several AUVs. Any of these would be prohibitively expensive and not likely to be funded by NOAA research programs, especially for a ‘thickness’ indicator that is not as informative as area or volume, for resource management.*

We agree with this general assessment. However, we were referring to the inclusion of dynamic covariates like wind speed directly within the geostatistical model, as alluded to in the following sentence. We have modified the text to help clarify (Lines 424-427).

*S—1, should the journal Environmental science and technology be capitalized here and elsewhere?*

Changed in references and in the Supporting Information file.

*Northern Gulf of Mexico is not a specific geographic location, and should be represented as …..northern Gulf of Mexico.*

Changed in Supporting Information file.

*(National Geophysical Data Ceter, 2001) should be ….Center….*

Changed in Supporting Information file.

*Additional details on the development of these equations is provided in Obenour et al., (2013). Should be ….are…*

Changed in Supporting Information file.

*SI—3 Rosette-mounted samplers, however, are on large rigs with other …suggest ‘frames’ rather than rigs…. ‘rig’ also used in manuscript text.*

Changed in Supporting Information file

*Figures SI—6	Summer-wide daily estimates of area, volume, and thickness with the 95% CI of area, and volume for 1997-2000	Question: why are area values daily and volume and thickness are continuous (if, I am reading this graphic correctly)?*

All of the estimates are daily, which we feel is nearly continuous given the time scales under consideration here. We just use different line types (solid, dashed, dotted) to help differentiate the three metrics. As the captions are correct, we don’t think changes are needed.

*Table S9c. Candidate variables used in Summer Average regressions (MR indicates Mississippi River, AR indicates Atchafalaya River, W indicates western shelf). Capitalize “River” If Mississippi and Atchafalaya rivers is used, ‘river’ is not capitalized as it is not a geographic location.*

Changed in Supporting Information file.
