# Supplementary material for: Trends and drivers of hypoxic thickness and volume in the northern Gulf of Mexico: 1985–2018
Source: PLoS One. 2024 Dec 5;19(12):e0302759. doi: 10.1371/journal.pone.0302759 (PMC11620451; doi:10.1371/journal.pone.0302759)
Supplement: Attachment — Submitted filename: Matli PONE Hypoxia thickness and volume nGoMx.docx [file pone.0302759.s004.docx]

Matli PONE Hypox thickness and volume of hypoxia nGoMx

Some thoughts came to mind as I re-read this manuscript, not particularly with the quality of the manuscript, but with some of the results. I can see why thickness is less tractable than area or volume because the thickness feature is a result of winds piling up water masses to the east at the time when Gulf coastal winds shift from the east to from the west. [Also noted in the last sentence of the abstract.] There are also summer conditions where winds from the north may prevail or from the south, or a switching in the prevailing winds during the research cruise. These several conditions would influence the volume (not so much the total) and thickness.

….on average, there is a seasonal lag in peak hypoxic volume and thickness compared to hypoxic area…..

Are there any suggestions about the formation of hypoxia beginning in the sediments with respiration of organic matter and then migrating up into the lower water column, or a combination of this along with the respiration of settled phytoplankton below the pycnocline?

Another reason that hypoxic volume may not be considered a standard metric for the nGoMx hypoxic zone is that the system is open on the westward and southern sides, or disjunct among two areas, and therefore more difficult to estimate.

Not important, and an editor will catch these, but a space before a period in L64.

L100 delete comma after et al. Same for line 134. This may be a punctuation issue for the journal. There is usually not a comma after et al. before a date. No more marked.

L217, meaning of ‘severe’	Again line 221, and elsewhere, L229, L348	Is this a relative value of volume or thickness?

Pointing out differences in the ‘east’ and ‘west’ portions of the study area is useful.

Conclusion, mixture of past and present tenses.
