# Supplementary material for: Trends and drivers of hypoxic thickness and volume in the northern Gulf of Mexico: 1985–2018
Source: PLoS One. 2024 Dec 5;19(12):e0302759. doi: 10.1371/journal.pone.0302759 (PMC11620451; doi:10.1371/journal.pone.0302759)
Supplement: Attachment — Submitted filename: PONE reviewer comments Matli and Obenour.docx [file pone.0302759.s002.docx]

PONE reviewer comments Matli & Obenouer

This analysis extends prior analyses of area and volume to include thickness as a metric with which to compare annual and monthly trends and to support resource management decisions. Thickness as a metric is more difficult to understand compared to area (a synthetic number) and volume (also a synthetic number) compared to thickness, which as a synthetic number, is more difficult to understand because it is compiled from multiple hydrographic profiles across a broad range of depths and geographic locations and represents a unified metric that seems less useful as a management tool, either in reduction of nutrient inputs or fishery-related resources.

There is also a mixture of geographically different monitoring cruises, especially differences between TAMU and EPA compared to each other and to LUMCON. Additionally, LUMCONT is a one- or two-day cruise to specific transects contained within the larger LUMCON MC station locations during the shelfwide cruises. It is not clear how these various hydrographic profiles become a monthly or annual metric for statistical analyses.

Many stated results, e.g., hypoxia is more likely to occur in July and August than in June, is already well known. Winds from the east in spring disperse nutrients and fresh water from the Mississippi River in the spring season support the phytoplankton biomass that eventually falls to the sea bed and is respired.

Many general terms, e.g., For volume, the corresponding results are 0, 2, 11, 10, and 1 years, respectively, indicating that peak volume tends to lag peak area, at least on average

…..taken together, these results suggest both patterns as well as aberrations in the seasonal….

There is little doubt that a tremendous amount of consistent, strong analytical methods, and a thorough understanding of the metrics provided are correct. These are trademarks of these co-authors.

There needs to be a more concise presentation of the results and their interpretation.

Small things that caught my eye:

Inconsistent use of ….hypoxia… as a noun, and …..hypoxic…..as an adjective ……hypoxia metrics, when considered along with other riverine inputs and meteorological factors in multiple regression models. Hypoxic volume,….

L36 …..the size of the hypoxic zone

L 63, Louisiana Universities Marine Consortium

L 80, thickness of hypoxia across the Gulf hypoxia region from May, second hypoxia should be hypoxic, and so on through the manuscript.

L 101, change to ….sampling frame….

Fig. 1, a mixture of font styles

L 172, multiple cruises in each of the years analyzed, but the geographic footprints of those cruises were quite different.

L 175 et seq. Selection of ‘seasons’ does not appear to be consistent, but rather the months in which there were cruises.

L186, Mississippi and Atchafalaya rivers

L 212, a lag of 12 days

L 261, is there a reference for this statement ROMS vs FVCOM?

L 300, …too little variance…

L 336, 2000 was a drought year, little stratification

L 354 – 356, these sentences are contradictory. Overall, these results suggest that most of the variability in hypoxic volume can be attributed to changes in hypoxic area. At the same time, variability in hypoxic thickness, which is only modestly correlated to area, is also important.

L 372-373, is there a conclusion here?

Fig. 5, symbols are difficult to see.

L 388-389, after reading this paragraph is there any merit to determining hypoxia thickness?

L 447, ability to constraint uncertainty…. Change to …constrain…or chloropigments

L 449 “…..Future work in improving this model can focus on incorporating covariates that capture short term changes in system conditions….” Reviewer, of these limitations, it would take dynamic measurements of conductivity, temperature, DO, Chla or chloropigments, possibly turbidity, through the water column with some type of profiler on a buoy that could capture the hypoxic thickness. Or, some type of AUV that can change position, lower raise probes, move to a new position, over some periodicity that would capture short-term variability suitable for the models. OR, several AUVs. Any of these would be prohibitively expensive and not likely to be funded by NOAA research programs, especially for a ‘thickness’ indicator that is not as informative as area or volume, for resource management.

S—1, should the journal *Environmental science and technology* be capitalized here and elsewhere?

Northern Gulf of Mexico is not a specific geographic location, and should be represented as …..northern Gulf of Mexico.

(National Geophysical Data Ceter, 2001) should be ….Center….

Additional details on the development of these equations is provided in Obenour et al., (2013). Should be ….are…

SI—3 Rosette-mounted samplers, however, are on large rigs with other …suggest ‘frames’ rather than rigs…. ‘rig’ also used in manuscript text.

Figures SI—6	Summer-wide daily estimates of area, volume, and thickness with the 95% CI of area, and volume for 1997-2000	Question: why are area values daily and volume and thickness are continuous (if, I am reading this graphic correctly)?

Table S9c. Candidate variables used in Summer Average regressions (MR indicates Mississippi River, AR indicates Atchafalaya River, W indicates western shelf). Capitalize “River” If Mississippi and Atchafalaya rivers is used, ‘river’ is not capitalized as it is not a geographic location.
